# Contribution of Vicine, Convicine, and New Derivatives to the Bitter Off-Taste of Fava Bean Proteins

**DOI:** 10.1021/acs.jafc.5c16349

**Published:** 2026-02-07

**Authors:** Luisa Irmer, Oliver Frank, Sebastian Minas, Silvia Schaefer, Johanna Hecht, Andreas Daschner, Maik Behrens, Corinna Dawid

**Affiliations:** † Chair of Food Chemistry and Molecular Sensory Science, TUM School of Life Sciences, 84503 Technical University of Munich, Lise-Meitner-Str. 34, D-85354 Freising, Germany; ‡ TUM Graduate School, TUM School of Life Sciences Weihenstephan, Technical University of Munich, Alte Akademie 8, D-85354 Freising, Germany; § 28362 Leibniz Institute for Food Systems Biology at the Technical University of Munich, Lise-Meitner-Str. 34, D-85354 Freising, Germany

**Keywords:** fava bean, protein, bitter taste, vicin, convicine, taste dilution analysis, plant-based alternatives, sensomics

## Abstract

This study employed activity-guided fractionation to identify the compounds that are responsible for the bitter off-taste of fava bean protein isolates and concentrates. UHPLC–ToF-MS and 1D/2D NMR experiments led to the identification of three known bitter compounds, vicine, convicine, and 3′-*O*-β-d-glucopyranosyl-L-DOPA. In addition, eight previously unknown vicine and convicine derivatives were identified. The bitter thresholds of the analytes were determined and found to be in the range of 0.10 to 1.44 mmol/L. To assess the taste contribution, the corresponding dose-overthreshold (DoT) factors were calculated, and it was shown that convicine with a DoT > 230 is playing a central role for the bitter off-taste. Furthermore, the analysis of fatty acids and their oxidation products suggests that linolenic-, linoleic-, and oleic acid directly contribute to the off-taste of fava bean protein. In addition, cell-based studies showed activation of bitter receptors TAS2R16 and TAS2R43 by vicine and convicine, respectively.

## Graphical abstract

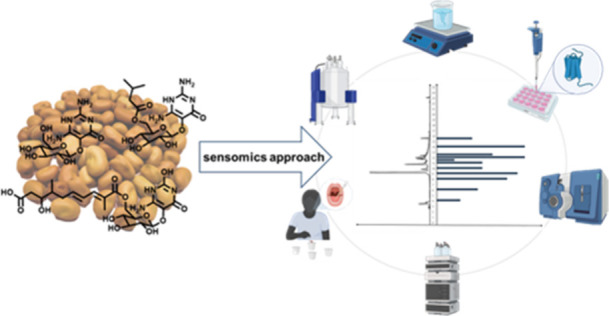

## Introduction

A declaration published by the World Health Organization^1^ (WHO) in 2021 states that a predominantly plant-based diet is associated with a healthy lifestyle and reduced risk of premature mortality.^2^ Limiting one’s consumption of processed meat helps reduce the risk of noncommunicable diseases (NCDs),^3–5^ such as cardiovascular diseases,^6,7^ cancer,^8^ and diabetes mellitus.^9^ NCDs are responsible for approximately 41 million deaths per year worldwide,^10^ and one in every five deaths is related to an unhealthy diet. In addition to its salutary effects on human health, a diet comprising primarily plant-based foods also has a beneficial impact on the global climate.^11,12^ Compared to animal-based products, the consumption of plant-based foods, such as vegetables/pulses, fruit, and grains, produces significantly lower levels of harmful greenhouse gases.^13^ Furthermore, the five-to-ten-times lower energy and water consumption and an 80% lower demand for agriculture land also emphasize the ecological benefits of a plant-based diet.^14^

Estimations are that by 2033 more than 20 million people in the European Union will be following a vegan or vegetarian/pescetarian diet.^15^ This already ongoing dietary shift has sparked a revolution in the European food market. To meet the growing demand for plant-based substitutes, innovative solutions based on plant-based foods must be found to create alternative products that meet consumers’ preferences. Pulses, such as soybeans or peas, are commonly used for this purpose, not only for their high protein content but also because they offer other environmental benefits.^16–18^ Although fava beans accounted for around 21% of the total grain legume area in Europe in 2020,^19^ they are still mainly used for animal feed^20^ and have limited use in the production of plant-based products for human nutrition. Despite fava beans’ high protein content and the good biological value of this protein—a protein digestibility corrected amino acid score (PDCAAS) value of 86.5%^21^—their products have so far found limited application in the food industry due to a bitter/astringent off-taste.^22,23^

Previous studies concerning the off-taste of legumes have indicated that a wide variety of sensory-active secondary metabolites may still be present in the protein fraction. Researchers attributed this presence to noncovalent interactions of taste-active compounds with proteins.^24–26^ Such protein-associated metabolites could be a major cause of the undesirable bitter taste of plant proteins. Potential metabolites discussed in that context include such compound classes as peptides^27^ and alkaloids.^28^ Furthermore, researches have demonstrated in pea protein, that fatty acids and fatty-acid-oxidation products that are formed either enzymatically or through autoxidation contribute to the bitter off-taste of protein isolates.^29,30^

The sensomics approach can be used to characterize the sensorially active compounds responsible for this undesired off-taste.^31^ To identify nonvolatile compounds from complex matrices, activity-guided screening methods are employed. These methods integrate instrumental-analytical techniques with human sensory analyses. The aim of this approach is comprehensive isolation and characterization of the key taste-active compounds. The localization of taste-active compounds by means of taste dilution analysis (TDA),^32^ facilitates the identification of the corresponding key tastants. This process is achieved through the application of nuclear magnetic resonance (NMR) and liquid chromatography–mass spectrometry (LC–MS) based experiments. To assess the sensory contributions of the identified analytes, dose-overthreshold (DoT) factors are determined, based on the concentrations of the stimuli in the respective food and their individual threshold values.^33^ Based on these values, reconstitution and omission experiments can then be carried out to clarify the additive and inhibitory effects.

While the off-taste of plant-based proteins derived from legumes, such as peas^29,30^ and rapeseed,^24^ has been extensively investigated using the sensomics approach, gaps remain in our knowledge of protein isolates and concentrates from fava beans. Very recently, Tuccillo et al.^34^ investigated metabolom variations in fava bean ingredients and were able to establish a relationship between off-flavors such as bitterness and some of the compounds that may be responsible for them, e.g., vicine and convicine among others. However, the authors concluded that, despite considerable efforts, there is a serious lack of information about the various bitter compounds found in foods such as fava proteins, leaving many questions unresolved, which we would like to answer in our study.

Its objective is to examine the exact molecular causes of the bitter off-taste of protein isolates and concentrates from fava beans. The first step is the isolation and structural identification of the key bitter tastants. Next, threshold values were determined by both human sensory analysis and receptor studies. By considering the corresponding dose–response relationships, we can then evaluate the contributions to the overall flavor can then be evaluated.

## Materials and Methods

### Chemicals

Deuterated dimethyl sulfoxide (DMSO-*d*_6_), l-tyrosine, ammonium acetate buffer, ammonium bicarbonate, and formic acid were commercially obtained from Merck (Darmstadt, Germany). Deuterated Uridine (uridine-*d*_2_) was purchased from Cayman Chemical Company (Michigan, USA). Organic solvents, including ethyl acetate, methanol, and *n*-pentane, were either HPLC grade (Merck, Darmstadt, Germany) or LC-MS grade (Honeywell, Seelze, Germany). All experiments were conducted using water purified with a Milli-Q Reference A+ system (Millipore, Schwalbach, Germany). Bottled water (Evian) was used for sensory analyses. Fava bean protein samples with a protein content ranging from 55 to 85% were provided by the food industry. The quantitation of the fatty acids and fatty acid oxidation products was performed according the method developed by Gläser et al.^30^

The bitter compounds used for the cell-based receptor analyses (vicine, convicine, vicine-6-*O*-isovalerate, and convicine-6-*O*-isovalerate) were either isolated from fava beans or, in the case of vicine, bought from Sigma-Aldrich (Taufkirchen, Germany). For the functional experiments, the stock solutions were formulated in the following assay buffer: 130 mM NaCl (Carl Roth, Karlsruhe, Germany), 10 mM Hepes (PAN BioTech, Aidenbach, Germany), 5 mM KCl (VWR), 2 mM CaCl_2_ (NeoFroxx, Einhausen, Germany) and 0.18% glucose (VWR), pH 7.4. The applied maximal compound concentrations were taken from a previous publication^35^ or limited by the available amount of substances.

### Cell Culture

A monolayer of HEK 293T-Gα16gust44 cells was cultivated in Dulbecco’s Modified Eagle Medium (DMEM) (Thermo Fisher Scientific, Schwerte, Germany), to which 10% heat-inactivated fetal bovine serum (FBS; Thermo Fisher Scientific), 1% L-glutamine solution (Sigma-Aldrich, Taufkirchen, Germany), and 1% penicillin-streptomycin solution (Sigma-Aldrich) were added. The cell culture plates were coated with 1 μg/mL poly-d-lysine (Sigma-Aldrich). Cell culture conditions were 37 °C, 5% CO_2_, and saturated air humidity.

### Transient Transfection

The cDNAs, including the entire coding regions of 26 human TAS2Rs, were available from previous studies.^36–38^ Transient transfections were conducted, as was done in previous studies.^39–41^ Briefly, HEK 293T-Gα166gust44 cells were seeded in 10 μg/mL poly-d-lysine (Sigma-Aldrich) coated clear-bottom 96-well plates, to reach a confluence of 60–70% the next day. For transient transfection, a mixture of 150 ng/well plasmid DNA and 0.3 μL/well lipofectamine 2000 (Invitrogen, Schwerte, Germany) was prepared. An empty vector (mock) was transfected as a negative control.

### Stable Cell Line

The stably transfected FLP-In T-REX 293-Gα16gust44-TAS2R43 was available from a previous study.^42^ The cells were seeded 1 day before measurement in 10 μg/mL poly-d-lysine (Sigma-Aldrich) coated clear-bottom 96-well plates, to reach a confluence of 90% the next day. To induce receptor expression, cells were treated with a supplemented DMEM solution of 0.5 μg/mL tetracycline 4h before measurement. Noninduced cells served as the control (mock).

### Calcium Imaging

The HEK293T–Gα16gust44 cells were loaded with the calcium-sensitive fluorescent dye Fluo4-am (Abcam, Cambridge, United Kingdom) in the presence of 2.5 mM probenecid (Sigma-Aldrich) and incubated at cell culture conditions for 1 h, as described previously.^39–41^ To investigate changes in fluorescence upon agonist stimulation, a FLIPR^TETRA^ system (Molecular Devices, San Jose, USA) was used. As a cell viability control, somatostatin 14 (end concentration of 100 nM; Bachem, Bubendorf, Switzerland) was applied.

### Terms/Abbreviations

The following terms/abbreviations are used in the text for the identified bitter compounds: vicine (**1**), convicine (**2**), vicine-6-*O*-isovalerate (**3**), convicine-6-*O*-isovalerate (**4**), (2*E*,4*E*)-8-hydroxy-2,7-dimethyl-2,4-decadiene1,10-dioic acid-6-*O*-vicine ester (**5**, HDDD-vicine), (2*E*,4*E*)-8-hydroxy-2,7-dimethyl-2,4-decadiene1,10-dioic acid-6-*O*-convicine ester (**6**, HDDD-convicine), divicine-5-O-(6′ → 1″)-*O*-β-d-diglucopyranoside (**7**), divicine-5-*O*-β-glucopyranosyl-(6′ → 1″)-*O*-α-xylopyranoside (**8**), isouramil-5-O-(3′ → 1″)-*O*-β-d-diglucopyranoside (**9**), isouramil-5-*O*-β-glucopyranosyl-(6′→1″)-*O*-α-xylopyranoside (**10**) and 3′-*O*-β-d-glucopyranosyl-l-DOPA (**11**).

### Sequential Solvent Extraction

An aliquot of the protein sample (300 g) was extracted three times with a methanol–water mixture (70:30, v/v, 1400 mL) by stirring for 30 min at room temperature, followed by vacuum filtration. The combined filtrates were removed from solvent in a vacuum at 40 °C and freeze-dried twice to give the methanol–water extractables (Extract 1). Afterward, the residue was extracted with methanol (1400 mL, Extract 2), then with ethyl acetate (1400 mL, Extract 3), and finally with *n*-pentane (1400 mL, Extract 4). To remove any residual solvent, the individual Extracts 1–4 were lyophilized twice and stored at −20 °C until required for comparative taste profile analysis. A scheme of the complete sample workup is displayed in Figure S1 (Supporting Information).

### Fractionation of the Extract with the Most Intense Bitter Off-Taste

Extract I, which exhibited the highest bitter taste activity, was dissolved in a methanol–water mixture (1/9, v/v) and separated into 13 fractions (F1–13) using the following gradient: 0 min, 0% B; 3 min, 0% B; 14 min, 50% B; 15 min, 50% B; 18 min, 70% B; 20 min, 100% B; 24 min, 0% B; 25 min, 0% B. For this purpose, the membrane-filtered mixture was injected onto an RP column (Synergi Hydro-RP, 250 × 21.2 mm, 4 μm, Phenomenex, Aschaffenburg, Germany). The effluent was monitored at 276 nm using a flow rate of 20 mL/min. The fractions obtained from multiple runs were combined and subsequently lyophilized twice prior to sensory analysis. The target compounds in Fractions 5 were subfractionated using a XBridge BEH Amide OBD Prep column (10 mm × 250 mm; Waters, Milford, USA), at a wavelength of 272 nm which led to eight subfractions (F5–1–8).To improve the separation, a column oven (Jasco, Gross-Umstadt, Germany) was used, which was set to 40 °C. Chromatography was performed using the following gradient: 0 min, 95% B; 2 min, 95% B; 3 min, 74% B; 19 min, 74% B; 21 min, 10% B; 23 min, 95% B. To ensure sufficient purity of the analyte in F5–6, it was purified on a Nucleodur HILIC column (250 × 4.6 mm, 5 μm; Macherey-Nagel, Düren, Germany) at a flow rate of 1 mL/min and UV detection at 272 nm. The gradient was maintained isocratically at 95% B for 3 min, then gradually decreased to 73% B within one min and then kept isocratically for 5 min. Within 0.10 min B was decreased to 70% and was kept isocratically for 11 min. For both the semipreparative and analytical fractionations of F5, solvent A was an aqueous ammonium bicarbonate buffer solution (0.2 M), while solvent B was acetonitrile.

The subfractionation of F8, which was carried out on a semipreparative Nucleodur π^2^ column (250 × 10 mm, 5 μm, Macherey-Nagel, Dürren, Germany), at a wavelength of 272 nm and a flow rate of 4.7 mL/min yielded three subfractions (F8–1–8–3) which could be obtained by the implementation of the following gradient: 0 min, 0% B; 3 min, 0% B; 4 min, 15% B; 5 min, 15% B; 5.10 min, 17% B; 14 min, 17% B; 16 min, 100% B; 18 min, 0% B; and 19 min, 0% B. Solvent B was composed of a mixture of acetonitrile and isopropanol (60:40, v/v), and solvent A comprised aqueous formic acid (0.1%). Due to the complexity of fraction 9, a division into F9a and F9b was implemented to facilitate an effective subfractionation. The isolation of taste-active compounds from Fraction 9a was carried out on a semipreparative HyperClone ODS column (250 × 4.6 mm, 5 μm; Phenomonex, Aschaffenburg, Germany) with a flow rate of 4.7 mL/min and UV detection at 276 nm. In total, four subfractions (F9a-1–F9a-4) were obtained using the following gradient: 0 min, 0% B; 3 min, 0% B; 8 min, 50% B; 9 min, 50% B; 9.5 min, 60% B; 11 min, 70% B; 14 min, 0% B; and 15 min, 0% B. Aqueous formic acid (0.1%) was used as solvent A, whereas acetonitrile was used as solvent B. To ensure the optimal purity of the key tastants for sensory analysis, F9a-3 was subjected to further purification via analytical HPLC chromatography with a flow rate of 1 mL/min. Subfraction F9a-3 was injected onto an RP column (250 × 4.60 mm, Synergi Polar-RP 80 Å, 4 μm; Phenomenex, Aschaffenburg, Germany) and the effluent was monitored at 281 nm. The target analyte in Fraction 9b was isolated using a C18 column (150 × 10 mm, Kinetex 100 Å, 4 μm Phenomenex, Aschaffenburg, Germany) leading to ten subfractions (F9b-1–F9b-10). The effluent was monitored at 272 nm. Solvent A was composed of aqueous formic acid (0.1%) and an acetonitrile-isopropanol mixture (60:40, v/v) was used as solvent B. The following gradient was applied: 0 min, 0% B; 3 min, 0% B; 3.5 min, 12% B; 15 min, 15% B; 18 min, 26% B; 19 min, 70% B; 20 min, 0% B; 21 min, 0% B.

Fraction 10 was subfractionated using a Luna PFP column (250 × 10 mm; 100 Å, 5 μm Phenomenex, Aschaffenburg, Germany). The flow rate was set to 5 mL/min, and the effluent was monitored at 276 nm, yielding three subfractions (F10-1–F10-3). The gradient started at 35% B and was kept isocratic for 24 min. Within four min B was increased to 60% and maintained for 2 min. Afterward solvent B was gradually decreased to 35% within 1 min. Solvent A consisted of aqueous formic acid (0.1%), and solvent B consisted of acetonitrile. The isolation of all target compounds in sufficient purity was confirmed by 1D NMR spectroscopy and UHPLC–ToF-MS experiments.

### Identification of Bitter Compounds in Subfractions F5-1, F5-3, and F5-7

The freeze-dried Fraction 5 was dissolved in water and membrane filtrated prior to analysis via HPLC. The subfractions F5-1, F5-3, and F5-7, which were collected from several semipreparative HPLC runs, were combined, freed from solvent in a vacuum, and lyophilized. Analyses via UHPLC–ToF-MS and NMR spectroscopy revealed vicine in F5-3, convicine in F5-1, and 3′-*O*-β-d-glucopyranosyl-l-DOPA in F5-7. The respective ^1^H and ^13^C NMR data are listed below. The data obtained is consistent with previous findings reported in the literature.^43^

Vicine (**1**) (Figure 1): LC–MS (ESI^–^): *m*/*z* 303.1012 [M – H^–^]. ^1^H NMR (600 MHz, DMSO-*d*_6;_ 300 K): δ 2.99–3.05 [m, 1H, H–C­(**4′**)], 3.06–3.12 [m, 1H, H–C­(**2′**)], 3.13–3.21 [m, 2H, H–C­(**3′/5**′****)], 3.39–3.46 [m, 1H, H–C­(**6a**′****)], 3.63–3.68 [m, 1H, H–C­(**6b**′****)], 4.24 [d, 1H, *J* = 7.8 Hz, H–C­(**1**′****)].^13^C NMR (150 MHz, DMSO-*d*_6;_ 300 K): 61.1 [C­(**6a**′**/6b**′****)], 69.7 [C­(**4**′****)], 72.9 [C­(**2**′****)], 75.9 [C­(**3**′****)], 77.6 [C­(**5**′****)], 107.4 [C­(**1**′****)], 113.2 [C­(**5**)]; 152.1 [C­(**4**)], 158.5 [C­(**6**)], 159.3 [C­(**2**)].

**Figure 1:**
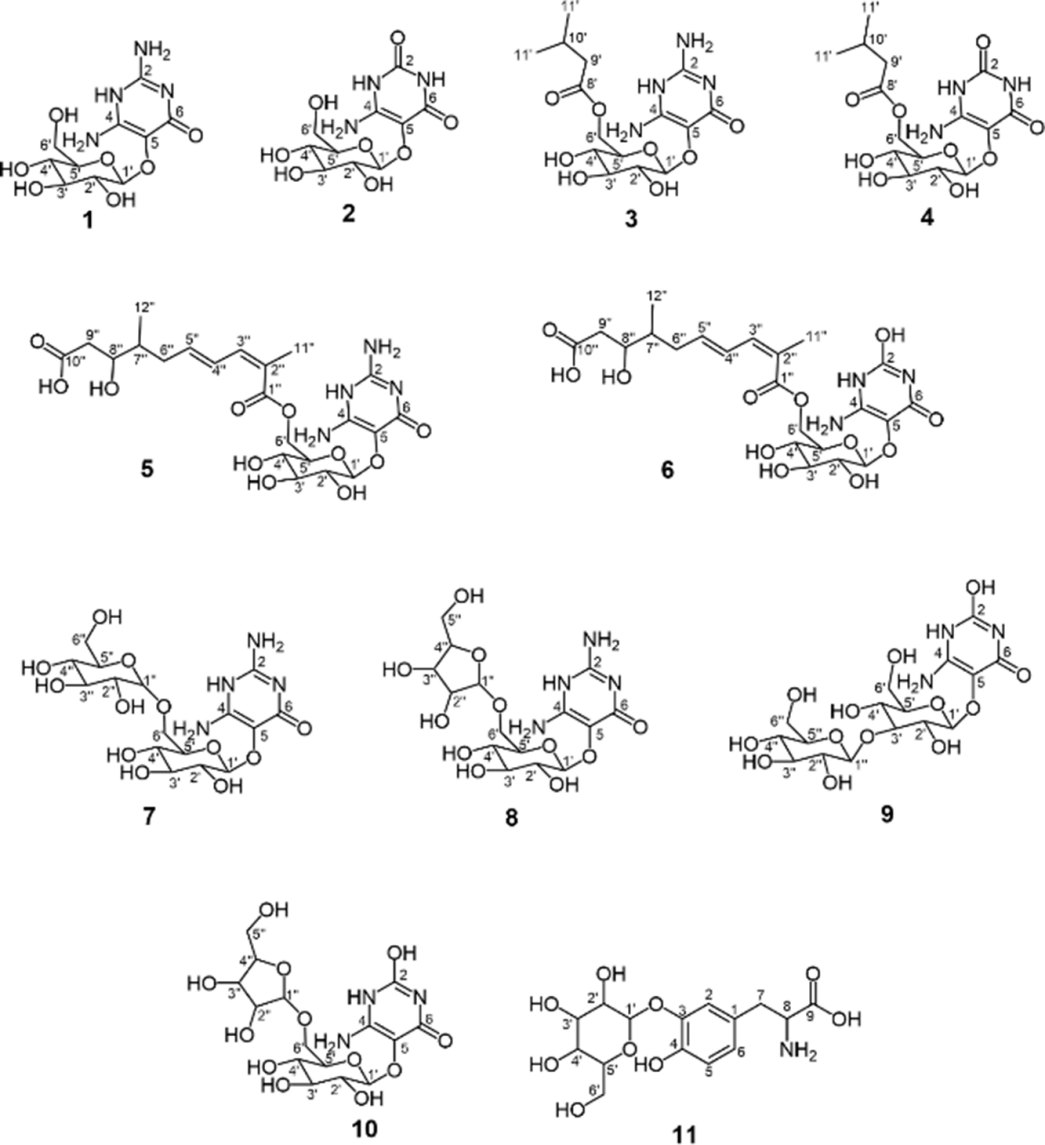
Chemical structures of vicine (**1**), convicine (**2**), vicine-6-*O*-isovalerate (**3**), convicine-6-*O*-isovalerate (**4**), HDDD-vicine (**5**), HDDD-convicine (**6**), divicine-5-*O*-(6′ → 1″)-*O*-β-d-diglucopyranoside (**7**), divicine-5-*O*-β-glucopyranosyl-(6′ → 1″)-*O*-α-xylopyranoside (**8**), isouramil-5-*O*-(3′ → 1″)-*O*-β-d-diglucopyranoside (**9**), and isouramil-5-*O*-β-glucopyranosyl-(6′ → 1″)-*O*-α-xylopyranoside (**10**), and 3′-*O*-β-d-glucopyranosyl-l-DOPA (**11**).

Convicine (**2**) (Figure 1): LC–MS (ESI^–^): *m*/*z* 304.0772 [M – H^–^]. ^1^H NMR (600 MHz, DMSO-*d*_6;_ 300 K):δ 2.99–3.06 [m, 2H, H–C­(**3**′**/5**′****)], 3.07–3.12 [m, 1H, H–C­(**2**′****)], 3.15–3.21 [m, 1H, H–C­(**4**′****)], 3.39–3.46 [m, 1H, H–C­(**6a**′****)], 3.66 [d, 1H, *J* = 12.1 Hz, H–C­(**6b**′****)], 4.34 [d, 1H, *J* = 7.8 Hz, H–C­(**1**′****)]. ^13^C NMR (150 MHz, DMSO-*d*_6;_ 300 K): δ 61.2 [C­(**6a**′**/6b**′****)], 69.9 [C­(**4**′****)], 72.9 [C­(**2**′****)], 75.9 [C­(**3**′****)], 77.5 [C­(**5**′****)], 107.3 [C­(**1**′****)], 110.2 [C­(**5**)]; 149.1 [C­(**4**)], 149.9 [C­(**2**)], 161.6 [C­(**6**)].

3′-*O*-β-d-Glucopyranosyl-l-DOPA (**11)** (Figure 1): LC–MS (ESI^–^): *m*/*z* [M – H^–^]. ^1^H NMR (600 MHz, DMSO-*d*_6;_ 300 K): 2.80–2.87 [m, 1H, H–C­(**7a**)], 2.96 [dd, 1H, *J* = 4.4/14.4 H–C­(**7b**)], 3.12–3.17 [m, 1H, H–C­(**3′**)], 3.26–3.29 [m, 2H, H–C­(**2**′**/4**′****)], 3.30–3.35 [m, 1H, H–C­(**5′**)], 3.42–3.47 [m, 1H, H–C­(**8**)], 3.47–3.52 [m, 1H, H–C­(**6a**′****)], 3.71–3.75 [m, 1H, H–C­(**6b**′****)], 4.70 [d, 1H, *J* = 7.5 Hz, H–C­(**1**′****)], 6.70 [d, 1H, *J* = 8.1 Hz, H–C­(**6**)], 6.74 [dd, 1H, *J* = 8.1/1.5 Hz, H–C­(**5**)], 7.06 [d, 1H, *J* = 1.5 Hz, 1H, H–C­(**2**)]. ^13^C NMR (150 MHz, DMSO-*d*_6;_ 300 K): δ 36.1 [C­(**7**)], 55.3 [C­(**8**)], 61.0 [C­(**6**′****)], 70.1 [C­(**3**′****)], 73.4 [C­(**2**′**/4**′****)], 76.0 [C**(2**′**/4**′****)], 77.5 [C­(**5**′****)], 102.2 [C­(**1**′****)], 115.7 [C­(**5**)], 118.1 [C­(**2**)], 124.1 [C­(**6**)], 127.6 [C­(**1**)], 145.2 [C­(**4**)], 145.7 [C­(**3**)], 170.3 [C­(**9**)].

### High-Performance Liquid Chromatography (HPLC)

For preparative HPLC, the system used was equipped with PU-2080 Plus pumps, a DG-2080-53 degasser, an MD-2010 Plus-type diode array detector (DAD; Jasco, Gross-Umstadt, Germany), and a Rheodyne injection valve (Rh 7725i type; Rheodyne, Bensheim, Germany). A HPLC system (Jasco, Gross-Umstadt, Germany) comprising a pump (PU-2080 Plus), a degasser (DG-2080-53), a detector (MD-2010 Plus-DAD), and an autosampler (2055 Plus) was used for analytical and semipreparative liquid chromatography. ChromPass software (Jasco, Gross-Umstadt, Germany) was used for data acquisition.

### Nuclear Magnetic Resonance Spectroscopy (NMR)

Sample analyses were performed either on a Bruker AV III spectrometer operating at a frequency of 400.13 MHz and equipped with a BBI probe at 298 K or on a Bruker AV Neo 600 MHz system equipped with a cryo TCI probe (Bruker, Ettlingen, Germany) at 300 K in DMSO-*d*_6_. The acquisition of 1D/2D NMR spectra was performed in either NMR tubes (5 mm × 178 mm, Z107374 USC tubes; Bruker, Faellanden, Switzerland) or NMR tubes (100 mm × 3 mm; Hilgenberg, Münnerstadt, Germany). Data acquisition and processing were done with TopSpin software 3.6.0 or 4.1.1 (Bruker, Ettlingen, Germany). Zero and first order phase correction were executed manually, and baseline correction was performed automatically via the *absn* command.

### Quantitative Proton NMR Analysis (qHNMR)

For purity and concentration determination of the target compounds (**1**–**11**) by ^1^H NMR, the 400 MHz spectrometer was calibrated using the ERETIC 2 tool based on the PULCON methodology, as previously described.^44^ l-tyrosine (4.09 mmol/L) was employed as an external standard. The specific resonance signals at δ 6.80 (m, 2H) and 7.11 ppm (m, 2H) were used for integration. A stock solution (600 μL) of each compound was prepared in DMSO-*d*_6_ and analyzed. The calculation of the analyte concentration was performed by using the ERETIC 2 software tool (TopSpin, Bruker, Rheinstetten, Germany). The determined values were used for quantitative analysis via UHPLC–MS/MS.

### Ultraperformance Liquid Chromatography–Time of Flight–Mass Spectrometry (UPLC–ToF–MS)

For the untargeted UHPLC–ToF–MS analyses of the compounds, a Waters Synapt G2-S HDMS mass spectrometer (Waters, Eschborn, Germany) in combination with an Acquity UPLC core system (Waters, Eschborn, Germany) for chromatographic separation, which was equipped with a sample manager, binary pump manager, and column oven (40 °C), was used. The samples were injected onto an Acquity BEH C18 column (150 mm × 2.1 mm, 130 Å; Waters, Eschborn, Germany). A flow rate of 0.4 mL/min was used with formic acid (0.1%) as the aqueous solvent and acetonitrile as the organic solvent. The gradient started at 5% B and was gradually increased to 100% B over eight min and maintained isocratic for 5 min. Data processing was performed using MassLynx V4.2 software (Waters, Eschborn, Germany).

### Ultrahigh-Performance Liquid Chromatography Triple Quadrupole Mass Spectrometry (UHPLC–MS/MS)

A QTRAP 6500 mass spectrometer (Sciex, Darmstadt, Germany), which was operated in a low molecular mass configuration connected to a Shimadzu Nexera X2 UHPLC (Shimadzu, Duisburg, Germany) equipped with an autosampler (SIL 30AC), a controller (CBM-20A), a degasser (DGU-20A5R), two pumps (LC-30AD), and a column oven (CTO-30A), was used. The parameters for the MS/MS system, which was operated in positive and negative electrospray ionization (ESI^+^/ESI^–^) modes, were as follows: Ion spray voltage: +4500 V/–4500; curtain gas: 35 psi; Gas 1:55 psi; Gas 2:65 psi; collision gas: medium; and source temperature: 500 °C. The software used for data acquisition was Analyst 1.6.3 (Sciex, Darmstadt, Germany).

For the quantitative analyses of compounds **1**–**5** and **11** by means of UHPLC–MS/MS, the individual MS/MS parameters were tuned first by directly infusing the purified reference compounds via a syringe pump and then optimized on the system described above while at least two specific mass transitions per analyte were recorded. All MS/MS analyses were conducted in the multiple reaction monitoring (MRM) mode. The analytes were injected onto an Acquity BEH Amide column (2.1 × 150 mm, 1.7 μm; Waters, Eschborn, Germany) and analyzed in the ESI positive (**1, 3**–**5**, and **11**) and negative (**2**) mode. For chromatography, aqueous ammonium acetate (5 mM, pH 2) was used as Eluent A and aqueous ammonium acetate (5 mM) in acetonitrile/water (95:5, v/v, pH 2) as Eluent B. The chromatographic separation was carried out at a flow rate of 0.4 mL/min at 40 °C, using the following gradient: 0.5 min, 100% B; 1 min, 96% B; 7 min, 96% B; 8 min, 40% B; 10 min, 40% B; 10.50 min, 100% B; and 15 min, 100% B.

To determine the concentrations of analytes **1**–**5** and **11** in the protein samples, an external calibration curve was used. For each analyte, a stock solution (100 μL) was prepared in water containing the target compounds in a range of 0.005–1690 μM. Therefore, stock solutions of vicine (0.9 mM), convicine (0.9 mM), vicine-6-*O*-isovalerate (1.52 mM), convicine-6-*O*-isovalerate (0.45 mM), HDDD-vicine (0.9 mM), and 3′-*O*-β-d-glucopyranosyl-l-DOPA (0.9 mM) were prepared in water. The exact concentration of the individual stock solutions was determined via qHNMR. These stock solutions were then diluted with water as follows: 1:2, 1:5, 1:10, 1:20, 1:50, 1:100, 1:200, 1:500, 1:1000, 1:2000, 1:5000, 1:10,000, 1:20,000, and 1:50,000. To receive an external calibration curve, the concentrations of the analytes were plotted against the respective peak area using linear regression. To compensate for potential workup losses, uridine-*d*_2_ (1 mg/mL) as an internal standard (IS) was added prior to sample preparation.

Two aliquots of each protein sample were weighed for the sample preparation of compounds **1**–**5** and **11**. Subsequently, 8 mL of the extraction mixture was added (for compound **1**, **2**, and **11**: 80 μL IS solution +7920 μL MeOH-water mixture (70/30, v/v); for compound **3**–**5**: 40 μL IS solution +7960 μL MeOH-water mixture (70/30, v/v)). The suspension was stirred for 30 min and then centrifuged (20 min, 3000 rpm), and the supernatant was collected and combined. Each sample was extracted twice with the internal standard added only during the first extraction step. The combined supernatants were freed from solvent in a vacuum, taken up in water (8 mL for compounds **1, 2**, and **11** and 4 mL for compounds **3**–**5**), and membrane filtrated prior to UHPLC–MS/MS analysis.

### Quantitation of Taste Active Lipids and Oxylipids via UHPLC–DMS–MS/MS

A triple determination was applied in order to quantitate the taste-active fatty acids and fatty acid oxidation products in the fava bean protein samples. The sample preparation was performed according to Gläser et al.^30^ As an internal standard, ^13^C_18_–Linoic acid and 18-Hydroxy oleic acid (0.5 mM in methanol) were added. The quantitation was performed using an external calibration curve. To each dilution step, the internal standards were added to achieve a final concentration of 0.005 mmol/L.

The MS/MS analyses were conducted by utilizing a QTrap 6500+ mass spectrometer. The instrument was equipped with a SelexION differential mobility separation device (DMS) cell (Sciex, Darmstadt, Germany) operating in the negative ionization (ESI^–^) mode. The parameter optimization was performed in reference to the methodology introduced by Gläser et al.^30^

### Sensory Analyses

#### General Conditions

Following a comprehensive risk assessment, 24 trained panelists (17 females and seven males, aged between 21 and 31), who consented to participate in the sensory experiments, were selected. All of the panelists had a central European background, which minimized the risk of glucose-6-phosphate dehydrogenase deficiency. Before any sensory analysis was done, the compound purity was ensured via ^1^H NMR spectroscopy. The samples were prepared in bottled water (Evian, Wiesbaden, Germany), and the pH value was adjusted to 5.5 with formic acid (0.1%). To prevent cross-modal interactions with odorants, the panelists wore nose clips.

#### Taste Profile Analysis

As a first step and to serve as a reference, one of the protein samples was subjected to a taste profile analysis. A portion of the sample (1.5 g) was suspended in 25 mL of water. The panelists were asked to evaluate the following six taste or organoleptic attributes on a scale of 0 (not perceivable) to 5 (strongly perceivable): *bitter, astringent, sour, sweet, salty,* and *umami*. The findings of this analysis served as a point of reference for subsequent gustatory experiments.

#### Comparative Taste Profile Sensory Analysis

The remaining eight protein samples were subjected to comparative taste profile sensory analysis. The panelists were asked to evaluate all samples according to a predefined scale of 0–5 based on predetermined reference values.

#### Taste Dilution Analysis

The fractions F1–13 from Extract I were dissolved three times above their natural concentration in bottled water (30 mL, pH 5.5) and then sequentially diluted 1:1 (v/v) with bottled water (pH 5.5). The diluted fractions were presented in ascending concentrations, starting with the highest dilution level, in the form of a Duo-Test. The panelists were asked to indicate the first perceivable difference between the sample and a negative control (bottled water, pH 5.5) and to identify the predominant taste attribute. This activity was used to determine the taste dilution (TD) factor for each individual fraction (Figure 2).

**Figure 2:**
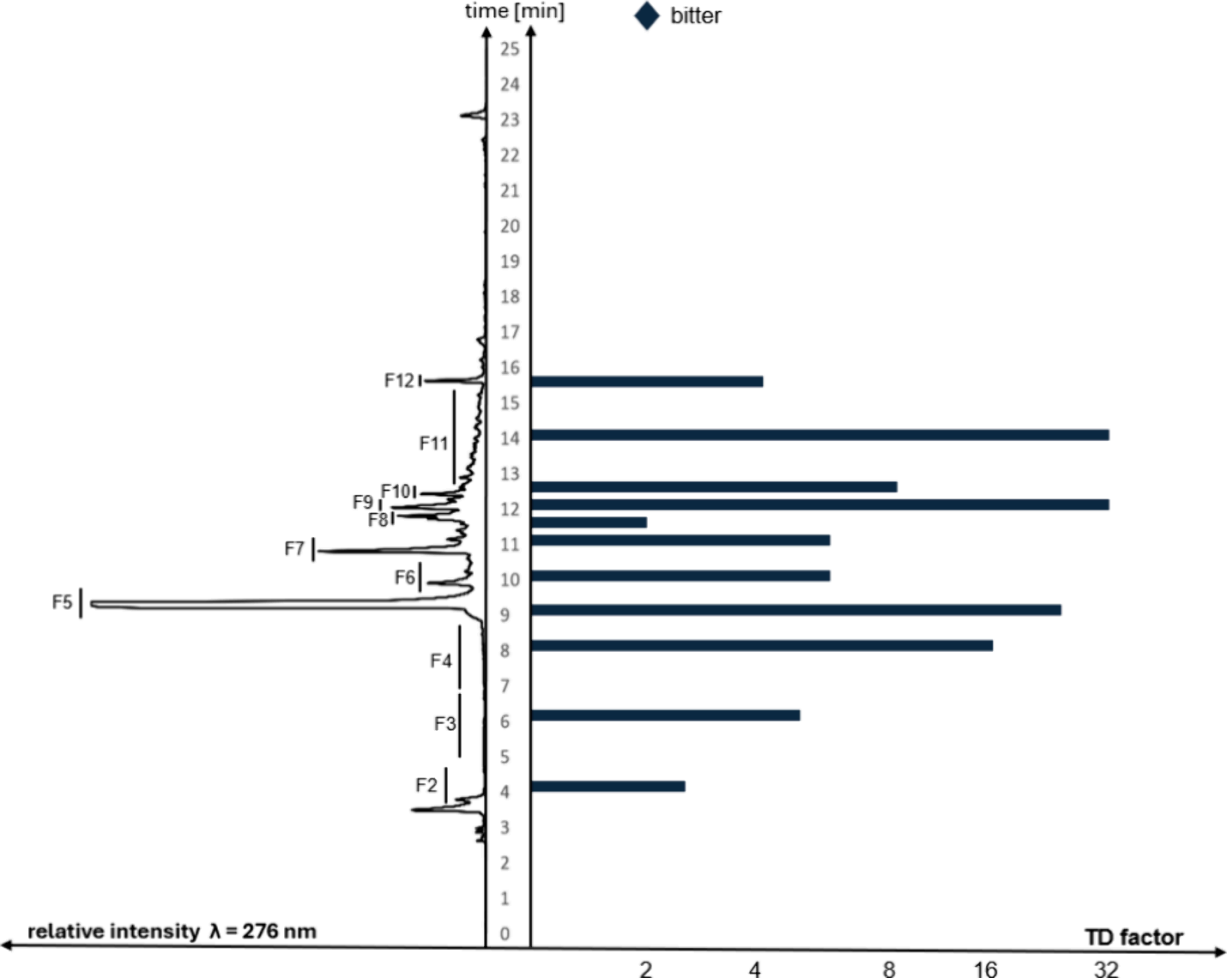
RP-HPLC chromatogram (left) of Extract 1 and (right) taste dilution analysis (TDA) with taste dilution (TD) values (right) of Extract 1.

#### Human Taste Recognition Threshold

The taste thresholds of the isolated compounds were determined in accordance with the literature.^45^ The test setup corresponded to a duo-test in which the panelists were asked to identify the dilution level at which the taste quality of the analytes could be detected from a blind control (bottled water, pH 5.5). Stock solutions for each analyte **1**–**5** and **11** (vicine—9.4 mmol/L; convicine—4 mmol/L; vicine-6-*O*-isovalerate—1.5 mmol/L; convicine-6-*O*-isovalerate—3 mmol/L; HDDD-vicine—2.4 mmol/L and 3′-*O*-β-d-glucopyranosyl-l-DOPA—4 mmol/L) were prepared with bottled water (pH 5.5) and successively (1 + 1) diluted.

#### Ethic Statement

The panelists were informed in advance, in writing, about the respective research questions and the possible risks of the study. The data collected were used exclusively for this study and were within its scope. The results were evaluated anonymously, so no conclusions could be drawn about individual participants. There was no obligation to participate, and participants could withdraw from the study at any time. Consent to data processing was voluntary and could be withdrawn at any time without giving reasons and without any disadvantages. Participants also had the right to request correction or deletion of their data. However, once the data had been anonymized, they could no longer be assigned to a specific person and could no longer be viewed, corrected, or deleted. Due to the risk assessment, pregnant employees were generally excluded from the sensory studies. The study was approved by the Ethics Committee of the Technical University of Munich (2023-36-S-KK).

## Results and Discussion

To achieve the goal of identifying the compounds responsible for the bitter off-taste of fava bean protein, the *sensomics* approach was applied. Therefore, nine protein samples obtained from the industry were presented to a trained sensory panel as part of a comparative sensory analysis. The protein sample with the highest average values for bitterness was judged with 2.5 on a scale from 0 (tasteless) to 5 (strong bitterness) and was subjected to further analyses. The sample was extracted with various solvents to determine in which solvent the most bitter compounds of the selected fava bean protein were located.

### Sequential Solvent Extraction

First, the selected protein sample was extracted with methanol/water (Extract 1), methanol (Extract 2), ethyl acetate (Extract 3), and *n*-pentane (Extract 4) in order of decreasing polarity. In addition, an insoluble residue was obtained. For the subsequent comparative sensory analysis, each fraction was freed from solvent in a vacuum and taken up in water in its natural concentrations. Extract 1 was rated as having the highest values across all taste attributes, followed by Extract 3. The panel determined a value of 3.0 for the taste attribute “bitter”, whereas Extract 3 was judged with a score of 1.8 for bitterness. For this reason, Extract 1 was subjected to further analyses by means of activity-guided fractionation, using the Taste Dilution Analysis (TDA; Figure 2).^32^

The comparison of the most bitter extract (methanol/water) with the most bitter protein sample (sample 5) showed that the panel rated the methanol/water extract as distinctively more bitter than sample 5. Presumably, a considerable number of bitter compounds are bound to the protein in a noncovalent manner and therefore cannot participate in taste perception. This indicates a key role of the protein–ligand interactions in the assessment of the intrinsic taste of proteins derived from plants. Moreover, these findings could potentially be crucial for the introduction of technological steps for the removal of taste-active compounds from protein fractions.

### Taste Dilution Analysis

To localize the compounds responsible for the bitter off-taste, Extract 1 was fractionated via preparative RP-HPLC into 13 subfractions, and the effluent was monitored at 276 nm (UV/vis). After the removal of the solvents in vacuum and two lyophilization steps, each fraction was taken up in equal amounts of water and subsequently subjected to a TDA.^32^ Therefore, each fraction was sequentially diluted 1:1 with bottled water (pH 5.5) and presented to the panel in the order of increasing concentration. The panelists were asked to evaluate each dilution step and compare the sample against a negative control (bottled water, pH 5.5). The dilution at which a first detectable difference in taste between the diluted sample solution and the negative control could be perceived is defined as the taste dilution (TD) factor. The highest TD factors for bitterness, with values of 24–32, were obtained for F5, F9, and F11. These fractions were therefore used for further isolation and identification of the bitter key compounds (Figure 2).

### Identification of Taste Active Compounds in Fraction 5

Due to its high TD factor of 24 for bitterness, high abundance, and comparatively low complexity, F5 was a promising fraction for further fractionation. Therefore, F5 was subfractionated by RP-HPLC, yielding eight subfractions F5-1–F5-8 (Figure S2, Supporting Information). Out of these, major fractions F5-1 and F5-3 were analyzed by UHPLC–ToF–MS in negative ionization mode (ESI^–^). In F5-3, a pseudo molecular ion ([M – H]^−^) with a mass-to-charge ratio (*m*/*z*) of 303.1012 was detected, which corresponds to an elemental composition of C_10_H_16_N_4_O_7_. In F5-1, a [M – H]^−^ with an *m*/*z* of 304.0772 was determined, most likely corresponding to the chemical formula C_10_H_15_N_3_O_8_.

Preliminary analysis of the *m*/*z* and the elemental composition suggest the presence of the analytes vicine (**1**) in F5-3 and convicine (**2**) in F5-1 (Figure 1). These amino pyrimidine glycosides are mainly found in fava beans (*Vicia faba* L.) and are classified as antinutritional factors.^46^ The structures of **1** and **2** could be unequivocally verified by analyzing the 1D/2D NMR data. The proton and carbon signals, *J*_H,H_ correlation in the COSY spectrum, ^1^ *J*_H,C_ correlation in the HSQC, and ^2,3^ *J*_H,C_ HMBC spectrum were found to align with the data reported in the literature.^43^

In subfraction F5-7 a *m*/*z* of 358.1911 in negative ionization mode was detected by UHPLC–ToF–MS analysis, which corresponds to the elemental composition C_15_H_21_NO_9._ For this compound, the signal pattern of a hexose could be observed in the ^1^H spectrum with corresponding correlations of the diastereotopic methylene group at 3.47–3.52 ppm (H–C­(6a′)) and 3.71–3.75 ppm (H–C­(6b″)) as an example. The signal resonating at 4.70 ppm with a coupling constant of 7.5 Hz was well in the line with the anomeric proton H–C(1′) of a β-configured glucose, and the other signals H–C­(2′-5′) also showed good agreement with glycosidically bound glucose. In addition to the glycosidic proton signals, a further spin system in the aliphatic region of the spectrum with a multiplet at 2.80–2.87 ppm, a doublet of doublet (dd) at 2.96 ppm and a multiplet at 3.42–3.47 ppm could be observed. Based on the ^1^ *J*_H,C_ of the HSQC spectrum these two signals could be assigned as the diastereotopic methylene group H–C­(7a/7b) and the methine proton H–C(8)). The analysis of the aromatic region of the ^1^H spectrum revealed three signals in the range of 6.70–7.06 ppm with the typical coupling pattern of a 3-fold substituted aromatic ring (H–C(2) ^4^ *J* = 1.5 Hz (d), H–C(6) ^3,4^ *J* = 8.1/1.5 Hz (dd), H–C(5) ^3^ *J* = 8.1 Hz (d)). The four quaternary carbon atoms C(1), C(3), C(4), and C(9) could be assigned by the HMBC correlation signals. Thereby, C(9), the carbon atom of carboxylic acid, exhibited the strongest paramagnetic shift at 170.3 ppm, whereas C(3) and C(4) showed the typical shifts of phenolic groups. The linkage of the glucose moiety to the aromatic ring was confirmed by the HMBC correlation of C(3) with the anomeric proton H–C(1′) of the glucose. Based on the 1D/2D NMR data, the mass-to-charge ratio and the fragmentation pattern in the UPLC–ToF–MS analysis (358.1911 → 196.0618) the acid residue of the glycoside could be identified as the nonproteinogenic amino acid l-3,4-dihydroxyphenylalanine (l-DOPA) and the analyte as 3′-*O*-β-d-glucopyranosyl-l-DOPA also known as l-DOPA-3-glucoside.

This compound, known from the literature, was first isolated from fava beans in 1961 by Nagasawa et al.^47^ The analyte was described as a glycoside of β-d-glucose and DOPA, using paper chromatography and colorimetry, among other methods. However, a more precise configuration could not be determined. In 1965, the group by Andrews et al.^48^ was able to identify the analyte as a 3-*O*-substituted derivative. In 2021, the presence of L-DOPA-3-glucoside in fava beans was confirmed by the implementation of high-resolution LC-MS measurements.^49^ Nevertheless, structural elucidation by NMR spectroscopy was performed for the first time as part of the present study.

The analysis of subfraction F5-8 via UHPLC–ToF–MS revealed a *m*/*z* of 465.1479 (ESI^–^) with an elemental composition of C_16_H_26_N_4_O_12_. The data of the 1D/2D spectra (Tables S1 and S2, Supporting Information) demonstrated the signal pattern of vicine as in F5-3. However, the ^1^H spectra revealed a further doublet with a typical coupling constant of *J* = 7.8 Hz resonating at 4.20 ppm, most likely corresponding to a second hexose moiety.

The characteristic coupling constants and chemical shifts, in conjunction with the *m*/*z* ratio, indicated the presence of a divicine-dihexose. In the HMBC spectrum optimized for ^2,3^ *J*_H,C_ couplings, the doublet at δ = 4.46 ppm showed a correlation to the quaternary carbon C(5) of the divicine moiety. Consequently, this doublet was assigned to the anomeric proton H–C(1′) of the hexose directly connected to the aglycone. The doublet at δ = 4.20 ppm was assigned to H–C(1″). The analysis of the TOCSY spectrum enabled the differentiation between the two hexose units. Based on that, the correlations observed in the COSY spectrum then provided a precise assignment of the protons within the two spin systems of the hexoses. For example, H–C(1′) showed correlations with the multiplet at 3.14–3.23 ppm with an intensity of three protons, which could be assigned to H–C(2′) and H–C(3′) of the hexose spin system directly bound to the aglycone and to H–C(3″) of the second sugar.

The signals at 2.92–2.96 ppm and 3.01–3.06 ppm, which belong to the second hexose based on the TOCSY correlations with H–C(3″), could be assigned to H–C(2″) and H–C(4″). The multiplet at 3.06–3.12 ppm represents a signal, which contains protons of both sugar moieties and could be assigned to H–C(4′) and H–C(5″). This signal showed a COSY correlation to H–C(5′) at 3.32–3.38 ppm. The assignment of the diastereotopic methylene groups of the two sugar units was achieved by analyzing the phase-sensitive HSQC spectrum. The doublets of doublets (dd) at 3.42 and 3.64 ppm showed cross peaks with the carbon atom resonating at 61.3 ppm, while the dd at 3.52 and 3.98 ppm showed a correlation signal with the carbon at 69.0 ppm.

The signal at 3.52 ppm showed a TOCSY correlation to H–C(1′) and was consequently assigned to H–C­(6a′) and H–C­(6b′), respectively. The two remaining signals could be assigned to H–C­(6a″) (3.42 ppm) and H–C­(6b″) (3.64 ppm) by TOCSY as well as HMBC correlation signals. The analysis of the HSQC spectrum enabled the assignment of the ^13^C signals to their respective carbon atoms, whereas the HMBC spectrum revealed key correlations for the linkage of the two sugars moieties. The anomeric proton H–C(1″) showed a correlation with the ^13^C signal at 69.0 ppm, which was assigned to C(6′) and clearly confirms the 6′ → 1″ link between the two sugars units. The clear identification of the second sugar as a β-glucose was achieved with the help of a *J-Res*olved experiment. The coupling constants between H–C(2) and H–C(3), H–C(3) and H–C(4) as well as H–C(4) and H–C(5) yielded values of 8.7, 9.0, and 9.2 Hz, respectively, clearly indicating an axial, axial position of these protons relative to each other.

Finally, the assignment of the missing quaternary C atoms was completed. As an example, the ^3^ *J*_H,C_ correlation of H–C(1′) to C(5) at 112.1 ppm could be observed in the HMBC spectrum. The remaining three carbon atoms of divicine, which demonstrated no correlations with neighboring protons, were assigned by comparison to the literature. Consequently, the ^13^C signal at 150.3 ppm was determined to represent C(4), and the signal at 157.8 ppm was assigned to C(6). Consequently, the remaining signal showing the most pronounced paramagnetic shift could be attributed to C(2). The analyte in F5-8 could be identified as divicine-5-*O*-(6′ → 1″)-*O*-β-d-diglucopyranoside (**7**).

The UHPLC–ToF–MS analysis of F5-5 revealed a *m*/*z* of 466.0813 in negative ionization mode (ESI^–^) and an elemental composition of C_16_H_25_N_3_O_13._ The 1D/2D NMR data showed a very similar signal pattern to the divicine-5-*O*-(6′ → 1″)-*O*-β-d-diglucopyranoside. In contrast to the molecule that has just been described, the aglycone/monoglucoside showed the typical signals and shifts of an isouramil/convicin unit, and in addition, there was a clear difference in the key correlation of the linkage between the two sugars. In the HMBC spectrum, the carbon signal C(1″) showed a correlation to the proton signal H–C(3′), conforming to a 3′ → 1″ linkage. The clear identification of the second sugar as β-glucose was also confirmed with the help of a *J-Res*olved experiment. Therefore, the target compound in F5-5 was identified as isouramil-5-O-(3′ → 1″)-*O*-β-d-diglucopyranoside (**9**) (Tables S3 and S4, Supporting Information).

In Fraction F5-4 and F5-6 the *m*/*z* from the UHPLC–ToF–MS (ESI^–^) analyses were at 436.1303 and 435.3921 indicating additional convicine and vicine diglycoside derivatives well in line with hexose-pentose sugar moieties. The elemental composition corresponded to C_15_H_23_N_3_O_12_ (F5-4) and C_15_H_24_N_4_O_11_ (F5-6). The NMR data confirmed that suggestion based on the 1D/2D spectra. The key linkage between the two sugar units of both compounds were at the 6′ position of the hexose and the 1″ position of the pentose. Accordingly, the two analytes could be identified as divicine-5-*O*-β-glucopyranosyl-(6′ → 1″)-*O*-α-xylopyranoside (**8**) (F5-6; Tables S5 and S6, Supporting Information) and isouramil-5-*O*-β-glucopyranosyl-(6′ → 1″)-*O*-α-xylopyranoside (**10**) (F5-4; Tables S7 and S8, Supporting Information).

*J*-RESOLVED NMR experiments were introduced for the identification of the pentose. The determination of the key coupling constants for isouramil-5-*O*-β-glucopyranosyl-(6′ → 1″)-*O*-α-xylopyranoside of ^3^ *J*_1″,2″_ = 3.6 Hz, ^3^ *J*_2″,3″_ = 9.0 Hz led to the elucidation of the terminal sugar as α-d-xylose. The *J*-values are well in line with data from the literature.^50^ One of the most abundant pentoses in legumes such as fava beans is xylose, which is most likely derived from nonstarch polysaccharides such as hemicellulose and is present in both soluble and insoluble fiber fractions. In its xylan form, xylose is present in high quantities in the hull of the beans.^51,52^ A similar compound has been identified and quantitated in the flowers of *Pueraria lobata*, a plant from the Fabaceae family. The analyte tectorigenin-7-*O*-β-d-xylopyranosyl-(1–6)-*O*-β-d-glucopyranoside has the same sugar moiety, consisting of a glucose and a xylose, which, exactly as in compounds **8** and **10**, show the same linkage between the hexose and the pentose. The ^1^H NMR signal patterns were very similar, which also underlies the identification of the xylose.^53^ Unfortunately, from the *J*-RESOLVED NMR spectra of divicine-5-*O*-β-glucopyranosyl-(6′ → 1″)-*O*-α-xylopyranoside only limited information can be drawn due to overlapping signals. Based on the strong structural similarities of analytes **8** and **10** the presence of the same sugar molecules can be safely assumed.

The presence of these vicine and convicine derivatives was suggested in a 2021 study published by Kowalczyk et al.^54^ This suggestion was based on detected mass-to-charge ratios via UHPLC–MS/MS. Nevertheless, to the best of our knowledge, the structures have not yet been elucidated via NMR spectroscopy and reported in the literature.

### Identification of Taste-Active Compounds in Fraction 9

Fraction 9 exhibited the highest TD-factor for bitterness, with a value of 32. The very complex fraction has been divided into F9a and F9b after determination of the TD factor. Initially, F9a was subjected to further subfractionation via semipreparative RP-HPLC, leading to four subfractions F9a-1–F9a-4 (Figure S4, Supporting Information). The main compound eluting in F9a-3 was analyzed via UHPLC–ToF-MS and 1D/2D NMR spectroscopy. UHPLC–ToF–MS in the ESI mode showed a pseudomolecular ion ([M – H]^−^) at a *m*/*z* of 387.1503. This is well in line with elemental composition C_15_H_24_N_4_O_8_.

Analysis of the 1D/2D NMR data (Table 1) revealed two sets of signals within the ^1^H spectrum. One consisted of six protons, with signals resonating between 3.05 and 4.36 ppm, which were assigned to a sugar moiety. The chemical shift of the doublet at 4.30 ppm, with a characteristic coupling constant of 7.9 Hz, is typical for β-configured glucose. The three signals of the other set between 0.90 and 2.20 ppm showed strong correlations in the H,H–COSY spectrum; the doublet at 0.90 ppm with an integral of six was assigned to two isochronic methyl groups H–C(11′). The multiplet at 1.98 ppm was assigned to the methine proton H–C(10′). In the COSY spectrum, H–C(10′) showed ^3^ *J*_H,H_ correlations to the doublet at 0.90 ppm (H–C(11′)) and to another doublet at 2.20 ppm (H–C(9′)) and a coupling constant of 7.1 Hz. This signal with an integral of two was assigned to the methylene group H–C(9′). This structural feature most likely fits an isovaleric acid moiety.

**Table 1:** Assignment of ^1^HNMR Signals (600 MHz, DMSO-*d*_6_, 300 K) of Vicine-6-*O*-isovalerate (**3**) and Convicine-6-*O*-isovalerate (**4**)

	[ppm]		I	M		*J* [Hz]		gs-COSY
H at relevant C-atom	3	4	3 and 4	3	4	3	4	3 and 4
H–C(11′)	0.90	0.90	6	d	d	6.7	6.7	H–C(10′)
H–C(10′)	1.98	1.97	1	hept	hept	6.8	6.8	H–C(11′), H–C(9′)
H–C(9′)	2.20	2.17–2.20	2	d	m	7.1		H–C(10′)
H–C(2′/4′)	3.05–3.12	3.06–3.14	2	m	m			H–C(1′), H–C(3′), H–C(5′)
H–C(3′)	3.17–3.23	3.17–3.24	1	m	m			H–C(2′), H–C(4′)
H–C(5′)	3.41–3.47	3.40–3.47	1	m	m			H–C(3′), H–C(5′)
H–C(6a′)	3.99–4.05	4.00–4.07	1	m	m			H–C(5′), H–C(6′b)
H–C(1′)	4.30	4.41	1	d	d	7.9	7.9	H–C(2′)
H–C(6b′)	4.36	4.35	1	dd	dd	2.1/12.0	1.9/12.0	H–C(5′), H–C(6′b)

The ^13^C spectrum (Table 2) showed a total of 24 carbon atoms resonating between 22.1 and 172.3 ppm. The assignment of the ^1^ *J*_C,H_ correlations via the HSQC spectrum revealed 19 carbons connected to protons via ^1^ *J*_H,C_ couplings and five quaternary carbon atoms. Four of these could be assigned to the aglycon of vicine.^43^ The missing quaternary atom was the most paramagnetically shifted carbon of the ^13^C spectrum, resonating at 172.3 ppm. Analysis of the HMBC spectrum showed correlations of this carbon atom with H–C(9′) and H–C(10′) and could be assigned to the carbonyl group of isovaleric acid moiety C(8′). The linkage between vicine and the isovaleric acid was confirmed by the key correlation of the diastereotopic methylene group at H–C­(6a′/6b′) of the sugar moiety to the carbonyl carbon C(8′) via an ester bond. Taking all the spectroscopic data into account, compound **3** the major bitter compound in Fraction 9, was identified as vicine-6-*O*-isovalerate (Figure 1).

**Table 2:** Assignment of ^13^C NMR Signals (150 MHz, DMSO-*d*_6_, 300 K) of Vicine-6-*O*-isovalerate (**3**) and Convicine-6-*O*-isovalerate (**4**)

C-atom		[ppm]		heteronuclear H, C correlations		
3 and 4		3	4	^2,3,4^ *J* via HMBC		^1^ *J* via HSQC
C(11′)	CH_3_	22.1	22.1	CH(10′), CH_2_(9′), CH_3_(11′)	CH(10′), CH_2_(9′), CH_3_(11′)	CH_3_(11′)
C(10′)	CH	25.3	25.3	CH_3_(11′), CH_2_(9′)	CH_3_(11′), CH_2_(9′)	CH(10′)
C(9′)	CH_2_	42.5	42.6	CH_3_(11′), CH(10′)	CH_3_(11′), CH(10′)	CH_2_(9′)
C(6′)	CH_2_	63.2	63.1	CH(2′/4′), CH(5′)	CH(5′)	CH_2_(6′)
C(4′)	CH	69.7	69.6	CH(2’/4’), CH(3′), CH(5′), CH(6’a), CH(6’b)	CH(6a′), CH(6b′), CH(5′), CH(3′)	CH(4′)
C(2′)	CH	73.0	72.8	CH(3′), CH(6′a) CH(6′b)	CH(3′)	CH(2′)
C(5′)	CH	74.3	74.3	CH(1′), CH(2′/4′), CH(6′a), CH(6′b), CH(5′)	CH(1′), CH(6a′), CH(6b′)	CH(5′)
C(3′)	CH	75.8	75.6	CH(1′), CH(2′/4′), CH(5′)	CH(1′), CH(2′/4′), CH(5′)	CH(3′)
C(1′)	CH	107.7	106.6	CH(2′/4′), CH(5′)	CH(2′/4′), CH(5′)	CH(1′)
C(5)	C	113.7	110.1	CH(1′)	CH(1′)	
C(2)	C	152.0	149.3			
C(6)	C	158.7	161.1			
C(4)	C	159.1	150.1			
C(8′)	C	172.3	172.4	CH(6′a), CH(6′b), CH_2_(9′), CH(10′), CH_3_(11′)	CH(6′a), CH(6′b), CH_2_(9′), CH(10′)	

Since the presence of another convicine derivative was assumed in Fraction 8, the identification of the main compound was conducted, despite the comparatively low TD factor of 2 for the bitterness of F8. The dominant *m*/*z* ratio of 388.1224 in F8-3 (Figure S3, Supporting Information), determined by UHPLC–ToF–MS in negative ionization mode, confirmed the molecular formula C_15_H_23_N_3_O_9_ with a corresponding molecular mass of 389.14. F8-3 was further purified via semipreparative RP-HPLC, and the isolated compound **4** was analyzed by 1D and 2D NMR experiments.

An almost identical signal pattern to **3** was observed in the 1D and 2D NMR spectra, which suggests a strong structural similarity (Table 1). The chemical shifts of the spin system of the hexose protons H–C(1′) to H–C­(6a′/b′) were basically identical. Only a paramagnetic shift of the anomeric proton H–C(1′) could be detected, resulting in the doublet with a coupling constant of 7.9 Hz resonating at 4.41 ppm instead of 4.30 ppm, as observed for **3**. The signals for the isovaleric acid moiety showed the same multiplicities and chemical shifts; only minor differences in the coupling constants for the proton signals could be observed. The ^13^C signals of the glycoside moieties for **3** and **4** were also nearly identical (Table 2). Only the carbon atoms of aglycones C(2) and C(6) showed a different order of chemical shifts, which were well in line with an isouramil moiety. Based on the NMR data, combined with the results of the UHPLC–ToF–MS analysis, the compound eluting in F8-3 was identified as convicine-6-*O*-isovalerate (Figure 1). To the best of our knowledge, the structures of compound **3** and **4** have only been postulated based on UHPLC–ToF–MS analyses^49^ but not been elucidated via NMR spectroscopy in the literature yet.

### Identification of Taste-Active Compounds in Fraction 10

The TDA revealed fraction 10 as a bitter fraction with a TD factor of 8. The isolation of the taste-active compounds was performed on a pentafluorophenyl (PFP) column with the effluent being monitored at 276 nm (Figure S6, Supporting Information). The main compound eluting in subfraction F10-2 was analyzed via UHPLC–ToF–MS in negative ionization mode. A *m*/*z* value of 527.1992 (ESI^–^) was observed, along with a corresponding elemental composition of C_22_H_32_N_4_O_11_. Based on the mass fragmentation and the interpretation of the 1D/2D NMR, another vicine derivative was expected. The untargeted UHPLC–ToF–MS analysis showed a characteristic fragmentation pattern for vicine (*m*/*z* 303.1012/141.1312).

The analysis of the ^1^H NMR (Table 3) spectrum showed six protons resonating between 3.5 and 4.5 ppm. These signals could be assigned to the pyranose protons of the sugar moiety of vicine. The doublet at 4.34 ppm, which was assigned to H–C(1′), showed a coupling constant of 8.0 Hz—well in line with a β-configured glucose. Furthermore, the ^1^H spectrum of aglycone showed three signals in the aromatic region between 6.13 and 7.11 ppm. Because of the typical coupling constants of 11.4/15.0 Hz these signals were assigned to the protons H–C(3″), H–C(4″) and H–C(5″). The coupling constants and the chemical shifts most likely correspond to a *trans-*configuration of the double bond.

**Table 3:** Assignment of ^1^HNMR Signals (600 MHz, DMSO-*d*_6_, 300 K) of HDDD-Vicine (**5**) and HDDD-Convicine (**6**)

	[ppm]		I	M		*J* [Hz]		gs-COSY
H at relevant C atom	5	6	5 and 6	5	6	5	6	5 and 6
H–C(12″)	0.81	0.79	3	d	d	6.9	6.8	H–C(7″)
H–C(7″)	1.52–1.62	1.56–1.62	1	m	m			H–C(6a″), H–C(6b″), H–C(8″), H–C (12″)
H–C(11″)	1.87	1.86	3	d	s	0.90		H–C (3″)
H–C(6a″)	1.96–2.04	1.96–2.01	1	m	m			H–C(5″), H–C(6b″), H–C(7″)
H–C(9a″)	2.15–2.23	2.20–2.26	1	m	m			H–C(8″), H–C(9b″)
H–C(9b″)	2.23–2.35	2.27–2.34	1	m	m			H–C(8″), H–C(9a″)
H–C(6b″)	2.23–2.35	2.27–2.34	1	m	m			H–C (5″), H–C(6a″), H–C(7″)
H–C(2′)	3.09–3.15	3.09–3.17	1	m	m			H–C(1’), H–C(3′)
H–C(4′)	3.09–3.15	3.09–3.17	1	m	m			H–C(3′), H–C(5′)
H–C(3′)	3.21	3.22	1	t	t	8.9	8.9	H–C(2′), H–C(4′)
H–C(5′)	3.45–3.52	3.44–3.50	1	m	m			H–C(4′), H–C (6a′), H–C(6b′)
H–C(8″)	3.76–3.81	3.78–3.83	1	m	m			H–C(7″), H–C (9a″), H–C(9b″)
H–C(6a′)	4.05–4.11	4.09–4.15	1	m	m			H–C(5′), H–C(6b′)
H–C(1′)	4.34	4.43	1	d	d	8.0	7.9	H–C(2)
H–C(6b′)	4.40–4.45	4.37–4.41	1	m	m			H–C(5′), H–C(6a′)
H–C(5″)	6.13–6.20	6.12–6.19	1	m	m			H–C(4″), H–C (6a″), H–C(6a″)
H–C(4″)	6.40	6.39–6.46	1	dd	m	11.4/15.0		H–C(3″), H–C(5″)
H–C(3″)	7.11	7.01	1	d	d	11.4	11.4	H–C(4″), H–C(11″)

The COSY spectrum confirms the correlation between H–C(4″) and H–C(3″) as well as H–C(5″) with H–C(4″). Additionally, H–C(3″) showed a correlation with a doublet at 1.87 ppm (3H), which could be assigned to the methyl group H–C(11″). The coupling constant of 0.9 Hz indicates a ^4^ *J*_H,H_ coupling between the methyl group H–C(11″) and H–C(3″), enabled by the allyl position. The other eight proton signals were observed in the aliphatic region of the ^1^H NMR spectrum. The correlations of these signals in the COSY spectrum suggest an alkane unit involving the protons H–C­(6a″/6b″), H–C(7″), H–C(8″), and H–C­(9a″/9b″) attached to two methyl groups H–C(11″) and H–C(12″). The paramagnetic shift of the proton H–C(8″) at approximately 3.80 ppm indicates its direct proximity to an electron-withdrawing substituent. In the absence of any additional correlations apart from those observed to H–C(7″) and H–C(9″), the presence of a hydroxyl group is indicated by taking into account the chemical shift and the mass-to-charge ratio.

The methyl group H–C(12″) with a chemical shift of 0.81 ppm showed a correlation to H–C(7″), clarifying its linkage to the alkane unit. The analysis of the HMBC spectrum showed a correlation of the methyl group H–C(11″) to H–C(3″), confirming the assumed ^4^ *J*_H,H_ long-range coupling. Moreover, the HMBC spectrum showed a correlation between H–C(5″) and the diasteriotopic protons H–C­(6a″/6b″), which confirms the linkage of the alkene and alkane units. The quaternary carbon atom at 124.5 ppm shows correlations to the protons H–C(3″), H–C(4″) and the methyl group H–C(1″) and was consequently assigned to C(2″) (Table 4).

**Table 4:** Assignment of ^13^C NMR Signals (150 MHz, DMSO-*d*_6_, 300 K) of HDDD-Vicine (**5**) and HDDD-Convicie (**6**)

C atom		[ppm]		heteronuclear H,C correlations		
5 and 6		5	6	^2,3,4^ *J* via HMBC		^1^ *J* via HSQC
C(11″)	CH_3_	12.6	12.6	CH(3″)	CH(3″)	CH_3_(11″)
C(12″)	CH_3_	13.9	13.9	CH(6a″), CH(6b″), CH(8)	CH(6a″), C(6b″), C(8″)	CH_3_(12″)
C(6″)	CH_2_	36.8	36.9	CH(4″), CH(8″), CH(12″)	C(4″), C(5″), CH(8″), CH(12″)	CH(6″)
C(7″)	CH	38.0	38.1	CH(6a″), CH(6b″), CH(9a″), CH(12″)	CH(5″), CH(6a″), CH(6b″), CH(8″), CH(9a″), CH(9b″), CH(12″)	CH(7″)
C(9″)	CH_2_	40.2	40.0	CH(8″)	CH(7″)	CH(9″)
C(6″)	CH_2_	63.8	63.7	CH(4′)	CH(4′)	CH(6′)
C(8″)	CH	69.7	69.5	CH(6a″), CH(6b″), CH(9a″), CH(9b″), CH(12″)	CH(6a″), CH(6b″), CH(9a″), CH(9b″), CH(12″)	CH(8″)
C(4′)	CH	69.7	69.7	CH(3′), CH(5′), CH(6a’), CH(6b’)	CH(3′), CH(4′), CH(6b′)	CH(4’)
C(2′)	CH	72.9	72.8	CH(3′)	CH(3′)	CH(2′)
C(5′)	CH	74.5	74.5	CH(3′), CH(4’), CH(6a’)	CH(4′), CH(6a′)	CH(5′)
C(3′)	CH	75.8	75.6	CH(4′), CH(5′)	CH(2′), CH(4′)	CH(3′)
C(1′)	CH	107.3	106.6	CH(2’), CH(3′)	CH(2′), CH(4′)	C(1′)
C(5)	C	113.8	109.9	CH(1′)	CH(1′)	
C(2″)	C	124.5	124.6	CH(3″), CH(4″), CH(11″)	CH(3″), CH(4″), CH(11′)	
C(4″)	CH	127.0	127.1	CH(3″), CH(6a″), CH(6b″), CH(11″)	CH(6a″), CH(6b″), CH(11″)	CH(4″)
C(3″)	CH	138.7	138.9	CH(4″), CH(5″), CH(11″)	CH(4″), CH(5″), CH(1″)	CH(3″)
C(5″)	CH	143.0	143.1	CH(3″), CH(6a″), CH(6b″)	CH(3″), CH(6a″), CH(6b″), CH(11″)	CH(5″)
C(4)	C	152.3	150.0			
C(6)	C	158.5	161.2			
C(2)	C	159.3	149.7			
C(1″)	C	167.7	167.8	CH(3″), CH(6a′), CH(6b′)	CH(3″), CH(6a′), CH(6b′) CH(11″)	
C(10″)	C	174.6	173.5	CH(8″), CH(9a″), CH(9b″)	CH(8″), CH(9a″), CH(9b″)	

The most deshielded carbon atoms showed shifts of 174.6 ppm (C(10″)) and 167.7 ppm (C(1″)) and could be assigned to the two carboxy groups present in the molecule. The diastereotopic protons of the glucose unit of vicine (H–C­(6a′/6b′)) show a correlation with C(1″). Accordingly, the dicarboxylic acid is linked to the glycoside by an ester linkage between the hydroxy group at C(6′) and the carboxylic acid at C(1″).

Upon consideration of all spectroscopic data, the compound in F10-2 was identified as (2*E*,4*E*)-8-hydroxy-2,7-dimethyl-2,4-decadiene1,10-dioic acid-6-*O*-vicine ester (**5**, HDDD-vicine) (Figure 1). A very similar *m*/*z* of 528.1823 (ESI^–^) with an elemental composition of C_22_H_31_N_3_O_12_ was detected in subfraction 9b-8 (Figure S5, Supporting Information) most likely corresponding to a HDDD-convicine derivative.

The analysis of the 1D/2D NMR spectra (Tables 3 and 4) demonstrated signal patterns similar to those of HDDD-vicine, thereby suggesting a high degree of structural similarity. The proton signals were found to be almost identical. Only small deviations in their chemical shifts, which are characteristic of convicine in comparison to vicine, were observed. These include the anomeric proton, which occurs at 4.43 ppm. Moreover, the chemical shift and order of the ^13^C atoms of the aglycone isouramil differ from divicine, which can be explained by differences in the electronic environment. Therefore, the carbon atoms were assigned in the following order: (5) at 109.9 ppm, C(2) at 149.7 ppm, C(4) at 150.0 ppm, and C(6) at 161.2 ppm. The 2D correlations in the COSY, HSQC and HMBC spectra are largely consistent. Following comprehensive analysis of all spectroscopic data, the compound was identified as (2*E*,4*E*)-8-hydroxy-2,7-dimethyl-2,4-decadiene1,10-dioic acid-6-*O*-convicine ester (**6**, HDDD-convicine). To the best of our knowledge, these compounds have not been reported in the literature. However, a study from 2006 concerning the isolation, identification, and determination of the antioxidant effect of glycolized carotenoid metabolites from the peels of *Cydonia vulgaris* fruits described the dicarboxylic acid (2*Z*,4*E*)-8-hydroxy-2,7-dimethyldeca-2,4-diene1,10-dioic acid in the form of a glycoside. The ^1^H and ^13^C NMR data as established by Fiorentino et al. is in good agreement with the spectroscopic data determined in the present study.^55^

### Characterization of Taste-Active Compounds in Fraction 11

F11 was assigned a TD factor of 32, indicating its high degree of taste-activity. For this very complex fraction, a multitude of substances have been postulated to be present, which may contribute to the intense bitter taste through additive effects. The nonpolar nature of the fraction suggested that fatty acids and oxylipids could potentially act as bitter agents in this context. This class of compounds has already been attributed a decisive role in the bitter taste of protein isolates in other legumes, such as peas.^29^ A methodology developed by Gläser et al.^30^ was utilized to confirm the presence of the following fatty acids and their oxidation products in the protein samples: ricinoleic acid, linoleic acid, linolenic acid, palmitic acid, stearic acid and oleic acid, 9,12,13-trihydroxydecadec-10-enoic acid (9,12,13-THOA), 9,10-dihydroxyoctadecanoic acid (9,10-DiHOME), (9*Z*,11*E*)-13-hydroxyoctadeca-9,11-dienoic acid (13-HODE), (10*E*,12*E*)-9-hydroxyoctadeca-10,12-dienoic acid (9-HODE), (10*E*,12*Z*)-9-hydroxyoctadeca-10,12-dienoic acid (9-HODE).

### Sensory Activity of Bitter Compounds

The identified bitter compounds **1**–**5** and **11** were subjected to taste threshold analyses. To ensure sufficient purity for sensory analyses, compounds **1**–**3** were further purified by semipreparative/analytical HPLC. The purity of the individual compounds was verified by qHNMR and UHPLC–ToF-MS. The bitter threshold analyses were performed by a group of 12 carefully selected panelists using a two-alternative forced choice (2-AFC) method. The bitter threshold concentrations of vicine and convicine were determined to be 1.44 and 0.18 mmol/L, respectively. In contrast, the values for the bitter threshold of the vicine/convicine derivatives vicine-6-*O*-isovalerateand and convicine-6-O-isovalerate were found to be 0.19 and 0.27 mmol/L, respectively. The bitter threshold determination of HDDD-vicine resulted in values of 0.1 mmol/L and for 3′-*O*-β-d-glucopyranosyl-L-DOPA in 0.35 mmol/L. No threshold analyses were possible for HDDD-convicine and all of the diglycoside derivatives due to limited sample amounts. Nevertheless, a distinct bitter taste was determined via the TDA and preliminary sensory tests.

Upon establishing the threshold values, a considerable divergence was noted in the perceptions of the members within the sensory panel. It is pertinent to note that the sensory analyses conducted as part of these studies were consistently carried out using the same pool of panelists. However, in the case of the threshold determination of vicine, six out of 12 panelists could not perceive a difference between the provided reference (water at pH 5.5) and the sample solutions in the two-alternative forced-choice test series, even at high starting concentrations of vicine (>9 mmol/L). These panelists are henceforth referred to as “non-tasters”. The remaining half of the panel reported a bitter taste. The threshold value is calculated based on the responses of the group designated as “tasters”. Because sample material of convicine and especially convicine-6-*O*-isovalerate was limited, only the tasters were subsequently used for the determination of the threshold values. Despite the absence of human threshold values for vicine and convicine in the literature, a recently published study by Karolkowski et al.^35^ demonstrated the interactions of these bitter substances at the cellular level through an in vitro receptor assay. Consequently, it was demonstrated that vicine activates the human bitter receptor TAS2R16. As was established in previous research, the TAS2R receptors exhibit a notable degree of genetic variation.^56^ However, in the study by Karolkowski et al., convicine showed no activation of any bitter receptors. In contrast to this result, convicine was, in the present human sensory study, identified as a potent bitter substance with a bitter threshold value of 0.18 mmol/L. For this reason, our own cell-based receptor studies were carried out with vicine/convicine and their isovaleric acid derivatives (**3** and **4**).

It is important to note that this distribution of tasters and nontasters was not observed for the threshold determination of vicine-6-*O*-isovalerate, which was tested with all 12 members of the panel. This suggests that the structural expansion, in the form of the isovaleric acid substituent, of compounds **3** and **4** results in the activation of additional bitter receptors.

### Cell-Based Receptor Studies

For the human oral cavity, it is well-established that all functional bitter taste receptors are expressed in sensory cells of taste buds.^57^ Hence, all compounds that activate one or several of these receptors will elicit a bitter taste. However, the expression of human bitter taste receptors is, by far, not limited to the oral cavity but extends to numerous tissues throughout the body. In these extra-oral tissues, the patterns of human TAS2Rs is far less homogeneous than in the oral cavity with important physiological consequences.^58^ To identify the TAS2Rs responsible for the bitterness of fava bean extract, 26 human TAS2Rs were screened with the amino pyrimidine glycoside derivatives **1**-**4**. The expression constructs coding for the 26 TAS2Rs were transiently transfected into HEK 293T-Gα16gust44 cells. After being loaded with the calcium-sensitive fluorescent dye Fluo4-am, the plates were transferred into a fluorometric imaging plate reader (FLIPR^tetra^). This device automatically applied the test substances and monitored fluorescence changes. The four compounds (**1**–**4**) were applied in two concentrations, 30 and 300 μM. Responses were evident only in TAS2R43 transfected (high-sensitive variant with Trp at position 35; accession number = NM_176884.2) cells stimulated with 300 μM convicine (Figure 3A). The limited availability of vicine-6-*O*-isovalerate prevented the application of higher concentrations despite the absence of signals at both concentrations. Vicine-6-*O*-isovalerate did not activate any receptor-transfected cells at a concentration of 30 μM. In contrast, a concentration of 300 μM induced strong receptor-independent fluorescence changes in mock-transfected cells. In addition, vicine did not activate any receptor-transfected cells. The absence of signals from mock-transfected cells even at the concentration of 300 μM, the substance availability, and a previous report about the vicine activation of TAS2R16 ^35^ led to this receptor being tested at higher concentrations after the initial screening (Figure 3B). Then dose-dependent responses of TAS2R16 expressing (accession number = NM_016945.3) cells at concentrations of 3 mM and above were observed, confirming previously published results.^35^ A full dose–response relationship could not be monitored due to receptor-independent signals at concentrations higher than 10 mM (Figure 3C). Since significant receptor-independent fluorescence changes were observed for the two isovaleryl derivatives, one can assume that the concentration ranges relevant for bitterness could not be tested in the cellular assay. For vicine, activation of TAS2R16 at the rather high concentrations of 3 and 10 mM was demonstrated. This concentration range corresponds closely with previously published results.^35^ Although in the study by Karolkowski et al., similar to the results shown here, the lack of signal saturation for the responses of TAS2R16 was documented, an EC_50_-concentration of 5.9 ± 1.2 mM was calculated. However, in light of the apparent lack of signal saturation also in the data from the present work, the exploration of an EC_50_ concentration was refrained, as even higher EC_50_ values are possible. The biggest difference between the study by Karolkowski et al. and the present findings relates to the substance convicine. Whereas Karolkowski and colleagues did not identify any TAS2R that reacted to this bitter compound, the present experiments demonstrated the activation of TAS2R43. This discrepancy can possibly be explained by the fact that a higher concentration of 300 μM was used in the screening carried out here, whereas Karolkowski et al. did not exceed 100 μM. At 100 μM, no receptor responses were observed in the assay performed here, not even for TAS2R43. It should be noted that the possibility of applying higher convicine concentrations compared to the study by Karolkowski et al. could be related to the stable inducible cell line used here. This cell line does not need to be transiently transfected with receptor constructs and, according to our own observations, therefore tolerates somewhat higher compound concentrations better.

**Figure 3:**
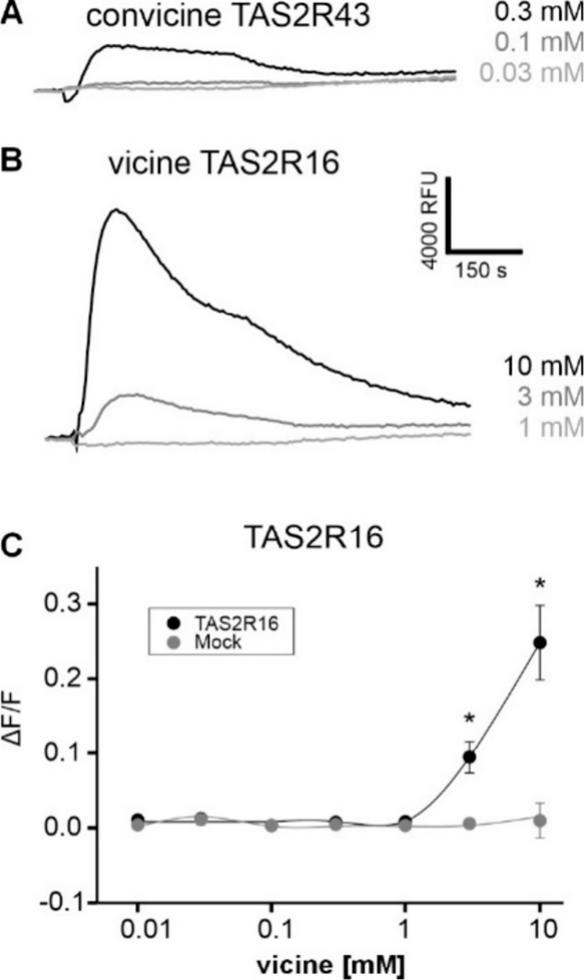
Human bitter taste receptors responding to vicine and convicine. (A) Expression of human bitter taste receptor TAS2R43, stably integrated in FLP-In T-REX 293-Gα16gust44-TAS2R43 cells, was induced with tetracycline before the experiment. Before stimulation with different concentrations (0.03, 0.1, and 0.3 mM) of convicine, cells were loaded with the calcium-sensitive fluorescent dye Fluo4-am. Representative traces of fluorescence changes induced by agonist stimulation are shown. (B) Human bitter taste receptor TAS2R16 was transiently expressed in HEK 293T-Gα16gust44 cells. Before stimulation with different concentrations (1.0, 3.0, and 10.0 mM) of vicine, cells were loaded with the calcium-sensitive fluorescent dye Fluo4-am. Representative traces of fluorescence changes are shown. Scale bar for (A) and (B) shown in (B). (C) Dose–response relationship of cells expressing TAS2R16 challenged with increasing concentrations of vicine. The relative changes in fluorescence are plotted onto the *y*-axis (Δ*F*/*F*) the vicine concentrations in mM are plotted onto the logarithmically scaled *x*-axis. Responses of receptor-expressing cells (TAS2R16, black) are shown along with negative controls (mock, gray). Asterisks indicate statistically significant fluorescence changes of receptor-expressing cells compared to mock controls.

### Quantitation of the Identified Bitter Compounds in Fava Bean Protein Samples and Calculation of the DoT Factors

In legumes, such as peas, it has been demonstrated that fatty acids and their oxidation products have a significant influence on the bitter off-taste of protein isolates.^29^ In order to assess their contribution to the off-taste in fava bean protein isolates and concentrates, the fatty acids and oxidation products identified in pea protein were also quantitated in fava bean protein samples. Within the fractionation of extract 1, they were located in F11 and concentrations in the range of 1.90 μmol/kg to 3.6 mmol/kg were detected. Regarding the oxidation products, 9,12,13-THOA, 9,10-DiHOME, 13-HODE, 9-HODE (ct), and 9-HODE (tt) were detected; however, the concentrations were comparatively low. Significantly higher concentrations were found for the identified fatty acids ricinoleic acid, stearic acid, palmitic acid, oleic acid, linolenic acid, and linoleic acid with the highest amounts found in linoleic acid followed by oleic acid.

The DoT factors were then calculated as the quotient of the concentration in the protein sample and the bitter threshold value, using the bitter threshold values known from the literature.^29,59–61^ In the case of (10*E*,12*Z*)-9-hydroxyoctadeca-10,12-dienoic acid, the threshold value of the 10*E*,12*Z* isomer was used, in accordance with the work of Gläser et al.^30^ Previous studies have demonstrated that the configuration of the double bond exerts a negligible influence on the bitterness threshold.^54^ In order to estimate the contribution to bitterness of compounds for which no threshold value is known, the lowest bitterness threshold of 0.08 mmol/L for fatty acid oxidation products was assumed for these compounds.

Only for linoleic acid, linolenic acid, and oleic acid DoT factors above 1 were determined, indicating a direct contribution to the bitter off-taste. Linoleic acid exhibited the highest DoT factor, with 3.7. The contribution of all other compounds to the overall bitterness is limited to synergistic or additive effects. The high concentrations of linoleic acid found in the protein samples are in good agreement with the concentrations found in fava beans. Studies have shown that linoleic acid is the main fatty acid and sometimes accounts for over 50% of the fatty acid composition.^62,63^

As part of this work, the concentrations of compounds **1**–**5** and **11** in nine protein samples were quantitated using UHPLC–MS/MS. For **1** and **2**, a concentration range between 8.9–75.0 μmol/g was found. In contrast, significantly lower values, between 0.11 nmol/g–8.12 μmol/g, were found for **3**–**5** and **11.** These results were then used to determine the DoT factors as a ratio of the concentration in the sample to the taste threshold of the respective tastants.^33^

The DoT factors of compound **2**, with a maximum of 233 and a minimum of 55, indicated that convicine is the most potent bitter compound of all of the analyzed compounds. In comparison, the DoT factors of vicine (**1**) in the protein samples were clearly lower, within a range of 12 to 50. In contrast, for analytes **3**, **4**, and **11,** DOT values between 1 and 11 were observed. For compound **5** the DoT factors ranged between 0.2 and 0.6. These findings indicate that the contributions of HDDD-vicine to the overall bitter off-taste of fava bean protein concentrates and isolates are mainly due to synergistic and/or additive effects, whereas a key contribution caused by **1**–**4** and **11** was confirmed as the DoT factor values were predominantly above one. Furthermore, the crucial role of **2** in the overall bitterness of fava bean protein has been confirmed.

In summary, this study is the first to determine the human bitter threshold concentrations of the pyrimidine glycosides vicine and convicine, which are ubiquitous in fava beans. Furthermore, eight vicine and convicine derivatives that have not been described in the literature before were identified by NMR and LC–MS experiments. The respective bitter thresholds of three of these analytes were determined. In addition, the influence of these compounds on the general bitter off-taste of fava bean protein was determined using the DoT factors. Furthermore, also linoleic acid, linolenic acid, and oleic acid were identified as key bitter tastants. Additionally, cell-based receptor studies were carried out, which demonstrated the activation of bitter taste receptor TAS2R16 by vicine and the activation of TAS2R43 by convicine.

The results show that all of the analytes described contribute significantly to the bitter off-taste of fava bean protein. Convicine is particularly important in this context, as it has a DoT value of over 230 in the most bitter protein sample and can therefore be described as “the most important bitter compound”. The findings of this study, therefore, make a significant contribution to the elucidation of the molecular causes of the bitter off-taste of the fava bean protein. With knowledge of the molecular nature of the most important bitter compounds, technological processes can now be developed, and targeted breeding research can be conducted to reduce or even eliminate these undesirable compounds in order to make the nutritionally valuable fava bean protein more attractive for human consumption.

## Supplementary Material



## Notes

Scheme for sample workup of the protein with the highest bitterness; ^1^H and ^13^C NMR data of the divicine- and isouramil-diglycosides; and HPLC chromatograms of the subfractionation procedure (PDF)
