# Supplementary material for: Contribution of Vicine, Convicine, and New Derivatives to the Bitter Off-Taste of Fava Bean Proteins
Source: J Agric Food Chem. 2026 Feb 7;74(6):5588–603. doi: 10.1021/acs.jafc.5c16349 (PMC12921848; doi:10.1021/acs.jafc.5c16349)
Supplement: Supplementary file 1 [file jf5c16349_si_001.pdf]

Supporting Information

Contribution of Vicine, Convicine, and new Derivatives to the Bitter Off-Taste of Faba Bean Proteins

Luisa Irmer[1], Oliver Frank[1*], Sebastian Minas[1], Silvia Schaefer[2,3], Johanna Hecht[1], Andreas Daschner[1], Maik Behrens[3] and Corinna Dawid[1,3*]

[1] Chair of Food Chemistry and Molecular Sensory Science, TUM School of Life Sciences, Technical University of Munich, Lise-Meitner-Str. 34, D-85354 Freising, Germany

[2]TUM Graduate School, TUM School of Life Sciences Weihenstephan, Technical University of Munich, Alte Akademie 8, D-85354 Freising, Germany

[3]Leibniz Institute for Food Systems Biology at the Technical University of Munich, Lise-Meitner-Str. 34, D-85354 Freising, Germany

---

* **Author to whom correspondence should be addressed:**

PHONE +49-8161/71-2910 (O.F.); -2901 (C.D.)

FAX +49-8161/71-2949

E-MAIL oliver.frank@tum.de; corinna.dawid@tum.de

Table of contents

Figure S1: Scheme for sample workup of the protein with the highest bitterness.

Table S1: Assignment of ^{1}HNMR signals (600 MHz, DMSO-*d*$_6$, 25 °C) of divicine-5-O-(6'→1'')-O-*β*-D-diglucopyranoside (7).

Figure S2: HPLC chromatogram of the sub-fractionation of F5 at λ = 272 nm (40 °C oven temperature).

Table S2: Assignment of ^{13}C NMR signals (150 MHz, DMSO-*d*$_6$, 25 °C) of divicine-5-O-(6'→1'')-O-*β*-D-diglucopyranoside (7).

Figure S3: RP-HPLC chromatogram of the sub-fractionation of F8 at λ = 272 nm.

Table S3: Assignment of ^{1}HNMR signals (600 MHz, DMSO-*d*$_6$, 25 °C) of isouramil-5-O-(3'→1'')-O-*β*-D-diglucopyranoside (9).

Figure S4: RP-HPLC chromatogram of the sub-fractionation of F9a at λ = 276 nm.

Table S4: Assignment of ^{13}C NMR signals (150 MHz, DMSO-*d*$_6$, 25 °C) of isouramil-5-O-(3'→1'')-O-*β*-D-diglucopyranoside (9).

Figure S5: RP-HPLC chromatogram of the sub-fractionation of F9b at λ = 272 nm.

Table S5: Assignment of ^{1}HNMR signals (600 MHz, DMSO-*d*$_6$, 25 °C) of divicine-5-O-*β*-glucopyranosyl-(6'→1'')-O-*α*-xylopyranoside (8).

Figure S6: RP-HPLC chromatogram of the sub-fractionation of F10 at λ = 276 nm.

Table S6: Assignment of ^{13}C NMR signals (150 MHz, DMSO-*d*$_6$, 25 °C) of divicine-5-O-*β*-glucopyranosyl-(6'→1'')-O-*α*-xylopyranoside (8).

Table S7: Assignment of ^{1}HNMR signals (600 MHz, DMSO-*d*$_6$, 25 °C) of isouramil-5-O-*β*-glucopyranosyl-(6'→1'')-O-*α*-xylopyranoside (10).

Table S8: Assignment of ^{13}C NMR signals (150 MHz, DMSO-*d*$_6$, 25 °C) of isouramil-5-O-*β*-glucopyranosyl-(6'→1'')-O-*α*-xylopyranoside (10).

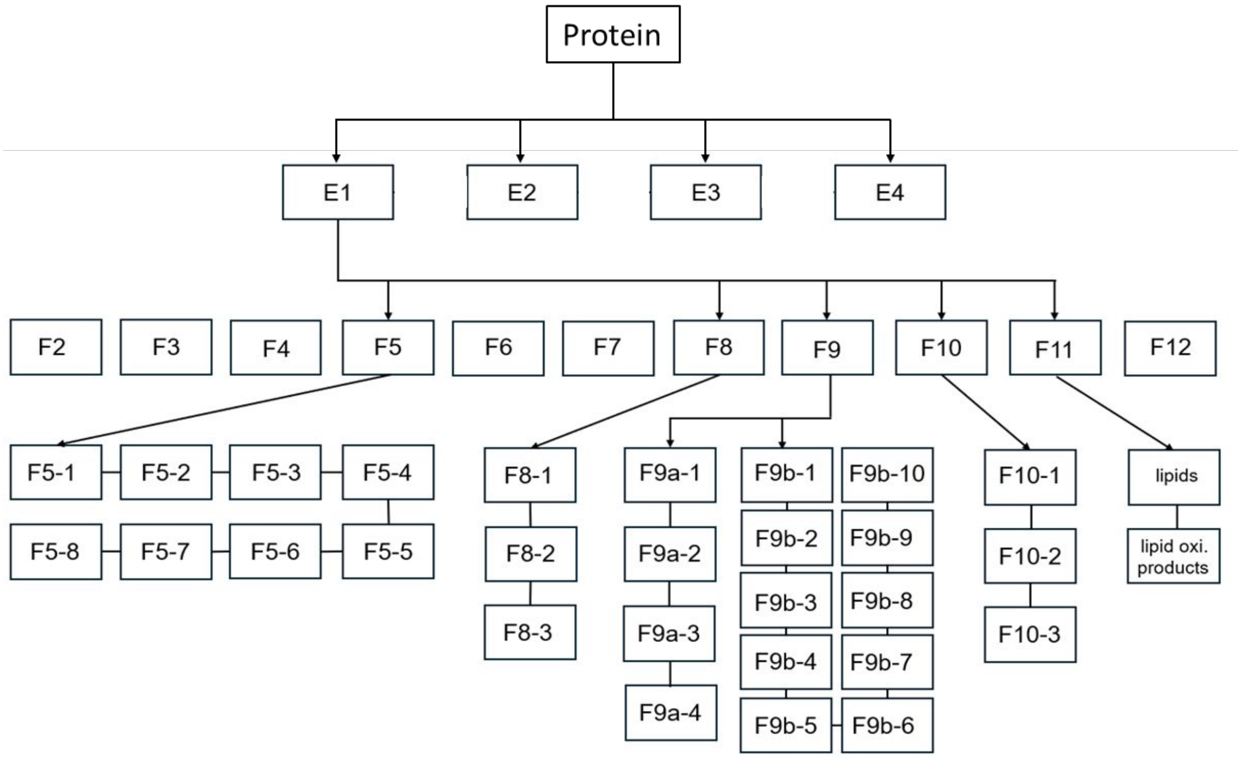

Figure S1: Scheme for sample workup of the protein with the highest bitterness.

Table S1: Assignment of ^{1}HNMR signals (600 MHz, DMSO-d_6, 25 °C) of divicine-5-O-(6'→1'')-O-β-D-diglucopyranoside (**7**).

**H at relevant C-atom**	**[ppm]**	**I**	**M**	***J* [Hz]**	**gs-COSY**
H-C(2'')	2.92-2.96	1	m		H-C(1''), H-C(3'')
H-C(4'')	3.01-3.06	1	m		H-C(3''), H-C(5'')
H-C(4')	3.06-3.12	1	m		H-C(3'), H-C(5')
H-C(5'')	3.06-3.12	1	m		H-C(4''), H-C(6a'')
H-C(2')	3.14-3.23	1	m		H-C(1'), H-C(3')
H-C(3')	3.14-3.23	1	m		H-C(2'), H-C(4')
H-C(3'')	3.14-3.23	1	m		H-C(2''), H-C(4'')
H-C(5')	3.32-3.38	1	m		H-C(4'), H-C(6a'), H-C(6b')
H-C(6a'')	3.42	1	dd	5.9/12.5	H-C(5''), H-C(6b'')
H-C(6a')	3.52	1	dd	7.1/11.3	H-C(5'), H-C(6b')
H-C(6b'')	3.64	1	dd	1.8/12.0	H-C(5''), H-C(6a'')
H-C(6b')	3.96-4.00	1	m		H-C(5''), H-C(6b'')
H-C(1'')	4.20	1	d	7.8	H-C(2'')
H-C(1')	4.46	1	d	7.6	H-C(1')

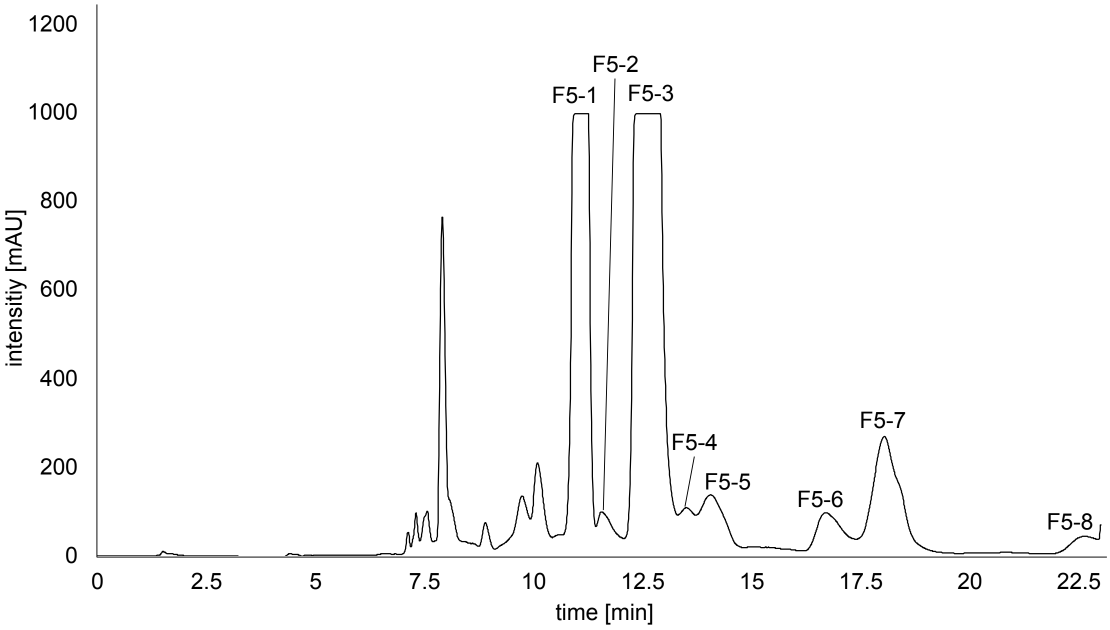

Figure S2: HPLC chromatogram of the sub-fractionation of F5 at λ = 272 nm (40 °C oven temperature).

Table S2: Assignment of ^{13}C NMR signals (150 MHz, DMSO-d_6, 25 °C) of divicine-5-O-(6'→1'')-O-β-D-diglucopyranoside (**7**).

**C-atom**		**[ppm]**	**heteronuclear H,C correlations**	
			$^{2,3,4}J_{C,H}$ via HMBC	$^{1}J_{C,H}$ via HSQC
C(6'')	CH_2	61.3	CH(4''), CH(5'')	CH(6'')
C(6')	CH_2	69.0	CH(4'), CH(5'), CH(1'')	CH(6')
C(4')	CH	70.1	CH(2'/3'), CH(6b')	CH(4')
C(4'')	CH	70.3	CH(3''), CH(5''), CH(6a''), CH(6b'')	CH(4'')
C(2'/3')	CH	73.2	CH(2'/3'), CH(4')	CH(2'/3')
C(2'')	CH	73.8	CH(3'')	CH(2'')
C(2'/3')	CH	76.1	CH(2'/3'), CH(5')	CH(2'/3')
C(5')	CH	76.3	CH(1'), C(4'), CH(6a'),	CH(5')
C(3'')	CH	76.7	CH(2'')	CH(3'')
C(5'')	CH	76.9	CH(1''), CH(2''), CH(4''), CH(6a''), CH(6b'')	CH(5'')
C(1'')	CH	103.5	CH(6a'), CH(6b'), CH(3''), CH(5'')	CH(1'')
C(1')	CH	105.8	CH(2'/3')	CH(1')
C(5)	C	112.1	CH(1')	
C(4)	C	150.3		
C(6)	C	157.8		
C(2)	C	163.5		

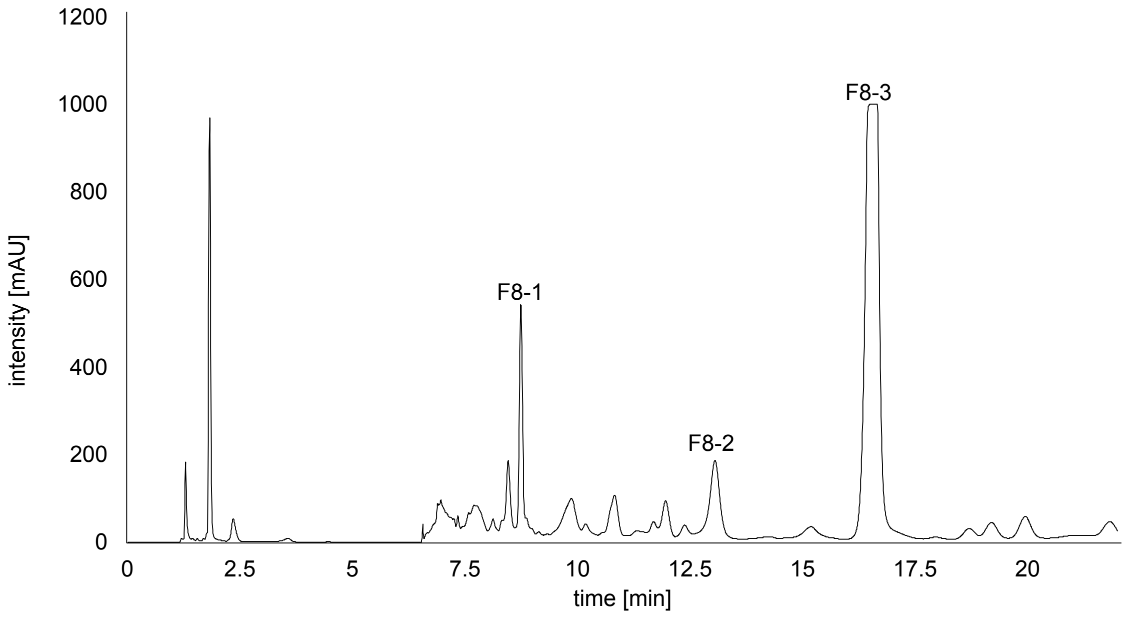

Figure S3: RP-HPLC chromatogram of the sub-fractionation of F8 at λ = 272 nm.

Table S3: Assignment of ^{1}HNMR signals (600 MHz, DMSO-d_6, 25 °C) of isouramil-5-O-(3'→1'')-O-*β*-D-diglucopyranoside (**9**).

H at relevant C-atom	[ppm]	I	M	*J* [Hz]	gs-COSY
H-C(2'')	3.00-3.06	2	m		H-C(1''), H-C(3'')
H-C(4'')	3.00-3.06	2	m		H-C(3''), H-C(5'')
H-C(3'')	3.12-3.20	3	m		H-C(2''), H-C(4'')
H-C(5')	3.12-3.20	3	m		H-C(4'), H-C(6a'), H-C(6b')
H-C(5'')	3.12-3.20	3	m		H-C(4''), H-C(6a''), H-C(6b'')
H-C(4')	3.24-3.30	1	m		H-C(3'), H-C(5')
H-C(2')	3.31-3.37	1	m		H-C(1'), H-C(3')
H-C(6a')	3.31-3.41	1	m		H-C(5'), H-C(6b')
H-C(6a'')	3.31-3.41	1	m		H-C(5''), H-C(6b'')
H-C(3')	3.43-3.50	1	m		H-C(2'), H-C(4')
H-C(6b')	3.62-3.70	1	m		H-C(5'), H-C(6a')
H-C(6b'')	3.62-3.70	1	m		H-C(5''), H-C(6a'')
H-C(1'')	4.38	1	d	7.8	H-C(2'')
H-C(1')	4.41	1	d	7.9	H-C(2')

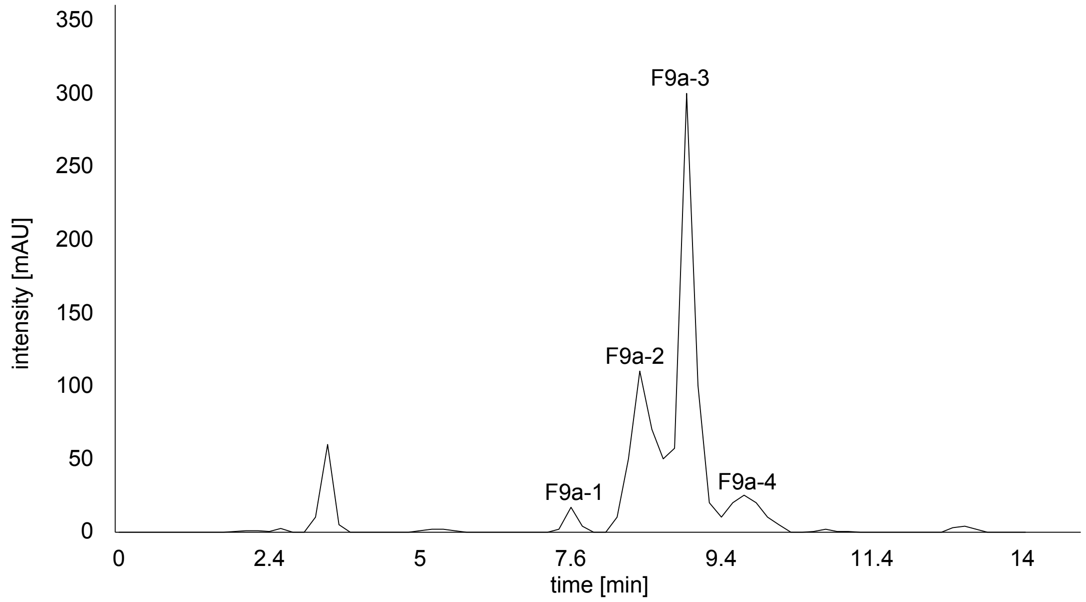

Figure S4: RP-HPLC chromatogram of the sub-fractionation of F9a at λ = 276 nm.

Table S4: Assignment of ^{13}C NMR signals (150 MHz, DMSO-d_6, 25 °C) of isouramil-5-O-(3'→1'')-O-*β*-D-diglucopyranoside (**9**).

C-atom		[ppm]	heteronuclear H,C correlations	
			$^{2,3,4}J_{C,H}$ via HMBC	$^{1}J_{C,H}$ via HSQC
C(6')	CH_2	60.9	CH(5')	CH_2(6'')
C(6'')	CH_2	61.1	CH(4'')	CH_2(6')
C(5')	CH	68.1	CH(3'), CH(4')	CH(5')
C(2''/4'')	CH	70.1	CH(3''),CH(5'')	CH(2''/4'')
C(2')	CH	72.2	CH(3')	CH(2')
C(2''/4'')	CH	74.1	CH(3''),CH(5'')	CH(2''/4'')
C(3''/5'')	CH	76.3	CH(2''/4'')	CH(3''/5'')
C(3''/5'')	CH	76.9		CH(3''/5'')
C(4')	CH	77.0		CH(4')
C(3')	CH	85.8	H-C(1'), H-C(2'), H-C(5')	CH(3')
C(1'')	CH	103.9	CH(3'), CH(2''/4'')	CH(1'')
C(1')	CH	107.0	CH(2')	CH(1')
C(4)	C	149.9		
C(2)	C	151.1		
C(6)	C	161.3		

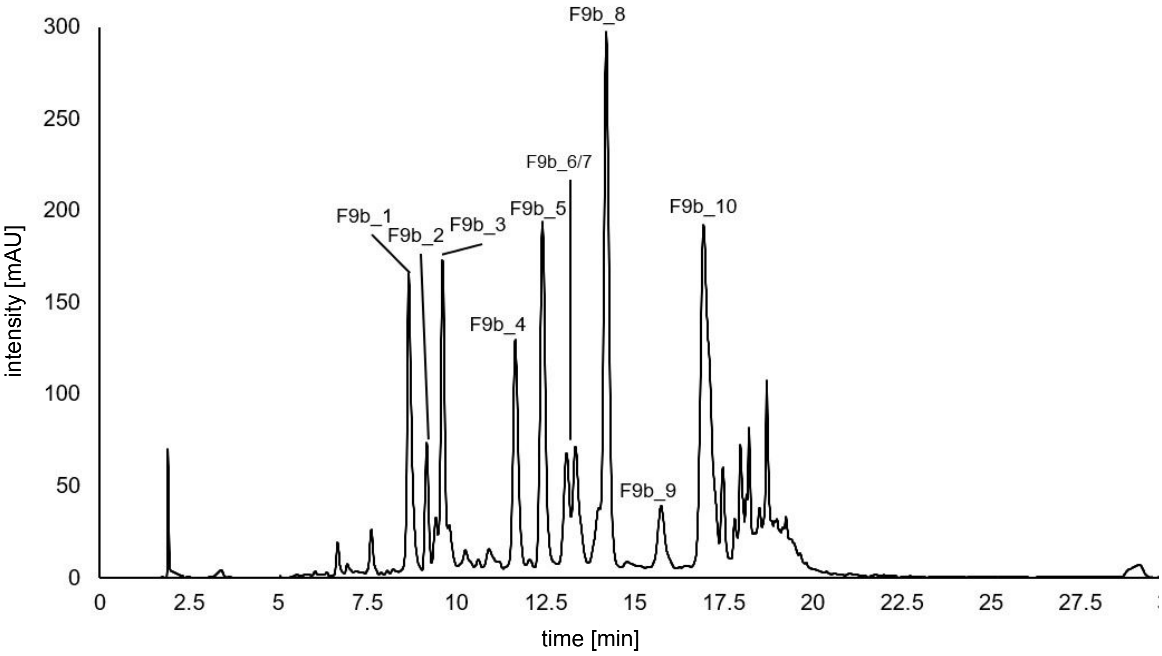

Figure S5: RP-HPLC chromatogram of the sub-fractionation of F9b at λ = 272 nm.

Table S5: Assignment of ^{1}HNMR signals (600 MHz, DMSO-d_6, 25 °C) of divicine-5-O-*β*-glucopyranosyl-(6'→1'')-O-*α*-xylopyranoside (**8**).

H at relevant C-atom	[ppm]	I	M	*J* [Hz]	gs-COSY
H-C(4')	2.96-3.07	1	m		H-C(3'), H-C(5')
H-C(2')	3.09-3.16	1	m		H-C(1'), H-C(3')
H-C(3')	3.16-3.23	1	m		H-C(2'), H-C(4')
H-C(2''/4'')	3.16-3.23	2	m		H-C(1''), H-C(3''), H-C(5'')
H-C(3'')	3.35-3.39	1	m		H-C(2''), H-C(4'')
H-C(5a''/5b'')	3.35-3.39	2	m		H-C(4'')
H-C(6a'/6b')	3.54-3.60	2	m		H-C(5')
H-C(1')	4.28	1	d	7.9	H-C(2')
H-C(1'')	4.62	1	d	3.4	H-C(2'')

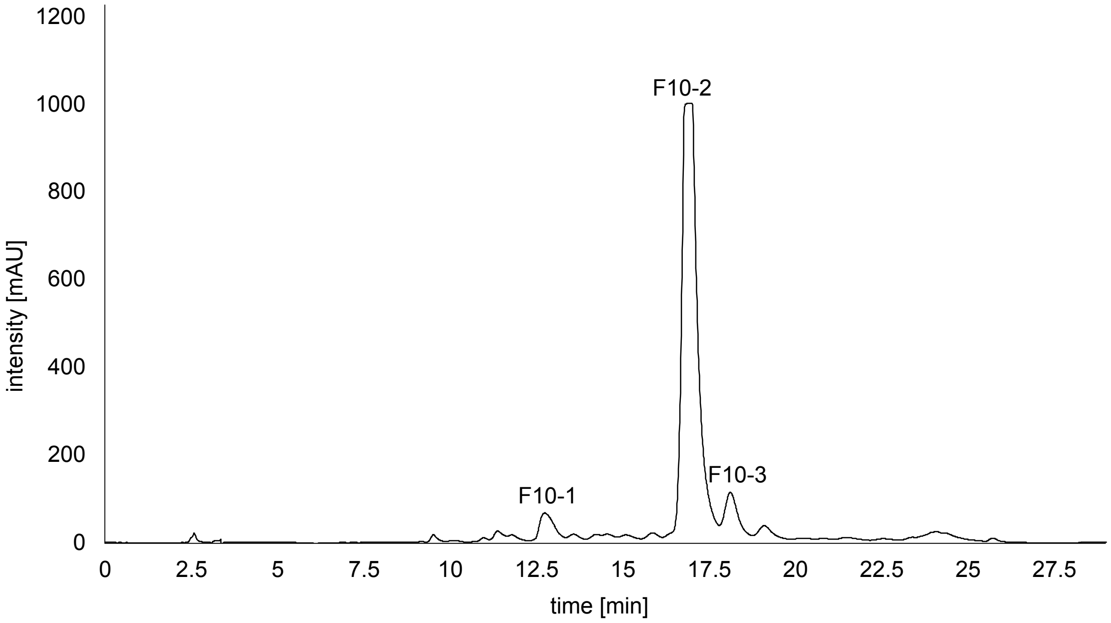

Figure S6: RP-HPLC chromatogram of the sub-fractionation of F10 at λ = 276 nm.

Table S6: Assignment of ^{13}C NMR signals (150 MHz, DMSO-d_6, 25 °C) of divicine-5-O-*β*-glucopyranosyl-(6’→1’’)-O-*α*-xylopyranoside (**8**).

**C-atom**		**[ppm]**	**heteronuclear H,C correlations**	
			$^{2,3,4}J_{C,H}$ via HMBC	$^{1}J_{C,H}$ via HSQC
C(5’’)	CH_2	61.7	CH(1’’), CH(3’’)	CH_2(5a’’/5b’’)
C(6’)	CH_2	66.2	CH(4’), CH(1’’)	CH(6a’/6b’)
C(2’’/4’’)	CH	68.2		CH(2’’/4’’)
CH(4’)	CH	70.3	CH(3’)	CH(4’)
C(2’’/4’’)	CH	71.9	CH(1’’), CH_2(5a’’/5b’’)	CH(2’’/4’’)
C(2’)	CH	72.8	CH(3’)	CH(2’)
C(3’’)	CH	73.2	CH(1’’), CH(2’’/4’’), CH_2(5a’’/5b’’)	CH(3’’)
C(3’)	CH	76.0	H-C(2’), H-C(4’)	CH(3’)
C(1’’)	CH	98.6	H-C(5a’’/5b’’)	CH(1’’)
C(1’)	CH	107.6	H-C(2’)	CH(1’)
C(5)	C	113.3	CH(1’)	
C(4)	C	152.4		
C(6)	C	158.4		
C(2)	C	159.3		

Table S7: Assignment of ^{1}HNMR signals (600 MHz, DMSO-d_6, 25 °C) of isouramil-5-O-*β*-glucopyranosyl-(6'→1'')-O-*α*-xylopyranoside (**10**).

H at relevant C-atom	[ppm]	I	M	*J* [Hz]	gs-COSY
H-C(3')	3.00-3.05	1	m		H-C(2'), H-C(4')
H-C(4')	3.07-3.11	1	m		H-C(3'), H-C(5')
H-C(2')	3.07-3.15	1	m		H-C(1'), H-C(3')
H-C(2'')	3.17-3.22	1	m		H-C(1'')
H-C(4'')	3.25-3.29	1	m		H-C(3''), H-C(5a''), H-C(5b'')
H-C(5a'')	3.29-3.31	1	m		H-C(4''), H-C(5b'')
H-C(3'')	3.35-3.39	1	m		H-C(2''), H-C(4'')
H-C(5b'')	3.38-3.41	1	m		H-C(4''), H-C(5a'')
H-C(5')	3.40-3.44	1	m		H-C(4'), H-C(6a'), H-C(6b')
H-C(6a')	3.55-3.59	1	m		H-C(5'), H-C(6b')
H-C(6b')	3.59-3.63	1	m		H-C(5'), H-C(6a')
H-C(1')	4.37	1	d	7.9	H-C(2')
H-C(1'')	4.63	1	d	3.5	H-C(2'')

Table S8: Assignment of ^{13}C NMR signals (150 MHz, DMSO-d_6, 25 °C) of isouramil-5-O-β-glucopyranosyl-(6'→1'')-O-α-xylopyranoside (**10**).

**C-atom**		**[ppm]**	**heteronuclear H,C correlations**	
			$^{2,3,4}J_{C,H}$ via HMBC	$^{1}J_{C,H}$ via HSQC
C(5'')	CH_2	62.0	CH(1''), CH(4''), CH(5a'')	CH_2(5a''/5b'')
C(6')	CH_2	66.8	CH(1''), CH(4')	CH_2(6a'/6b')
C(3')	CH	70.2	CH(5), CH(6a'), CH(6b')	CH(3')
C(4')	CH	70.2	CH(5), CH(6a'), CH(6b')	CH(4')
C(4'')	CH	70.2	CH(3''), CH(2'') CH(5b'')	CH(4'')
C(2'')	CH	72.1	CH(3''), CH(5b'')	CH(2'')
C(2')	CH	73.0		CH(2')
C(3'')	CH	73.4	CH(1''), CH(2''),CH(4''), CH(5a''/5b''),	CH(3'')
C(5')	CH	75.5	CH(1'), CH(4'), CH(6a'/6b')	CH(5')
C(1'')	CH	98.9	CH(6a'/6b'), CH(5a''), CH(5b'')	CH(1'')
C(1')	CH	107.1	CH(2)	CH(1')
C(5)	C	110.6	CH(1')	
C(4)	C	148.9		
C(2)	C	149.5		
C(6)	C	161.7		
